# Pace of aging, family environment and cognitive skills in children and adolescents

**DOI:** 10.1016/j.ssmph.2022.101280

**Published:** 2022-11-05

**Authors:** Gianmaria Niccodemi, Giorgia Menta, Jonathan Turner, Conchita D'Ambrosio

**Affiliations:** a University of Luxembourg, Luxembourg; b Luxembourg Institute of Socio-Economic Research (LISER), Luxembourg; c Luxembourg Institute of Health, Luxembourg

**Keywords:** Age acceleration, Epigenetic age, DNA methylation, Pace of aging, ALSPAC, Social class, Social environment

## Abstract

Pace of aging is an epigenetic clock which captures the speed at which someone is biologically aging compared to the chronological-age peers. We here use data from the Avon Longitudinal Study of Parents and Children (ALSPAC) to investigate the interrelation between the study children's parental social class at birth, and their pace of aging and cognitive skills measures in childhood and adolescence. We show that children from lower parental social classes display faster pace of aging and that the social class gradient in pace of aging is strongest in adolescence. About one third of this association can be explained by other socio-economic and demographic covariates, as well as life events. Similarly, study children's pace of aging manifests a negative association with their measures of cognitive skills in late adolescence only. This association becomes stronger as the contemporary pace of aging of the mother becomes faster. Our results seem to identify adolescence as the period of life when pace of aging, family environment and cognitive skills measures begin to interact.

## Highlights

• A negative gradient between paternal social class and child pace of biological aging gradually emerges during childhood.
• About one-third of this relationship can be explained by observable socio-economic covariates and life events.
• Faster biological aging in adolescence is associated with lower test-scores, more so if the mother is also aging faster.

## 1 Introduction

DNA methylation is the process of adding methyl groups (i.e., a cluster of 3 hydrogen atoms around a carbon atom) to DNA nucleotides. This mechanism is primarily used by cells to control and suppress gene expression, although it has multiple other functions, including a key role in X-chromosome inactivation in females (Chow et al., 2005; Sado et al., 2004). DNA methylation is mostly observed in CpG sites, DNA sequences in which cytosine and guanine are connected through a phosphate group.

In recent years, fluctuations in DNA methylation occurring with chronological age in some selected CpGs have been used to develop algorithms, known as *epigenetic clocks*, for the estimation of the so-called epigenetic age (Hannum et al., 2013; Horvath, 2013). With everyone aging at a different speed, epigenetic age estimated from these clocks is a powerful biomarker that predicts the biological capability and functional decline of individuals, as well as their morbidity and mortality.

Measures of DNA methylation *age acceleration* can be computed from the difference between biological age and chronological age. These have been shown to be positively associated with health outcomes such as obesity and indicators of metabolic syndrome (Horvath et al., 2014; Quach et al., 2017) and cardiovascular, cancer and all-cause mortality (Marioni, Shah, McRae, Chen, et al., 2015; Perna et al., 2016).

More recently, a second generation of clocks has been proposed with the identification of CpGs that track clinical measures, biomarkers of system integrity and biological ageing rather than chronological age only (Belsky et al., 2020; Levine et al., 2018). Remarkably, the clock developed by Belsky et al. (2020), named DunedinPoAm, acts as a *speedometer* for the rate of aging, as it is trained on longitudinal data on change in 18 biomarkers^1^ of organ system integrity, observed for individuals of the same age cohort between the age of 26 and 38. Thus, rather than being a clock that measures biological age, DunedinPoAm is a measure of the instantaneous pace of aging (PoA, from here onwards), namely the speed at which the individual is biologically aging compared to a reference population used for training PoA (in this case, the Dunedin Study participants).

Evidence from the literature suggests that early-life environment plays a role in affecting DNA methylation patterns. Fraga et al. (2005) show that the pattern of epigenetic changes in identical twins diverges as age increases. Similarly, Wong et al. (2010) show that DNA methylation differences appear in identical twins already in early childhood. Parental socioeconomic position might be a determinant of epigenetic aging: a few studies suggest that individuals with a more disadvantaged early-life socioeconomic status are epigenetically older in adulthood (Austin et al., 2018; Hughes et al., 2018), although such association is not detected by Lawn et al. (2018), McCrory et al. (2019), and Simpkin et al. (2017). Early-life adversities are also found to be associated with epigenetic age acceleration (Sumner et al., 2019), including exposure to violence (Jovanovic et al., 2017), traumas (Wolf et al., 2018) and sexual abuse (Lawn et al., 2018). Raffington et al. (2021a, 2021b) show that children and adolescents from families (and neighbourhoods) of low socioeconomic status exhibit a faster PoA as measured by DunedinPoAm.

Other studies also suggest the presence of associations between measures of epigenetic age acceleration and cognitive skills. Marioni, Shah, McRae, Ritchie, et al. (2015) report a link between greater age acceleration and worse cognitive ability in the elderly. Belsky et al. (2020) show greater cognitive decline over time for individuals with a faster DunedinPoAm PoA, based on their IQ scores measured at ages 7–13 and 45. Finally, Raffington et al. (2021b) find a negative association between the DunedinPoAm PoA and cognitive tests in children and adolescents.

In this paper we focus on PoA in children and adolescents, as measured by the DunedinPoAm algorithm, using English data from the Avon Longitudinal Study of Parents and Children (ALSPAC).^2^ We here investigate two separate research questions. First, we analyse the association between study children's parental social class, proxied by the paternal social class at birth, and their PoA at the ages of 7–9 and 15–19. Second, we investigate whether PoA at the same ages of 7–9 and 15–19 is associated with the study children's contemporary measures of cognitive abilities, including average school grades and IQ scores. In both cases we account for family environment, including pre-birth factors, socioeconomic position, and child characteristics.

We show that lower paternal social class when the study child is born is associated with higher child PoA at the age of 15–19. This association is nearly invariant after controlling for family environment, including pre-birth factors, and other indicators of the family's socioeconomic position, children characteristics and childhood adversities. At the age of 7–9 we do not observe consistent associations between the study child's paternal social class at birth and her PoA. This suggests that the paternal social class gradient in PoA may not appear until late adolescence. Similarly, late adolescence is the period of life when PoA manifests a negative association with measures of cognitive skills. Specifically, we find that the study children's PoA at age 15–19 is negatively associated with their nearly contemporary Key Stage 4 average grades, although we find evidence that about half of this association can be explained by our controls. In pre-adolescence, we do not observe any significant association between PoA (at the age of 7–9) and nearly contemporary Key Stage 1 average grades of the study children.

The current work is the first after Raffington et al. (2021a, 2021b) to study the association between PoA, family environment and cognitive skills measures. It differs from Raffington et al. (2021a, 2021b) as (i) we used blood DNA methylation data instead of saliva, improving on the reliability of the results as DunedinPoAm was initially developed on blood samples (Belsky et al., 2020), (ii) we exploited repeated measures of pace of biological aging observed at two points in time (i.e., childhood and adolescence) for the same individuals of the same age cohort and not cross-sectional data on a mixed sample of children and adolescents, (iii) we used a more diversified set of early-life variables that allowed us to assess their contribution to the associations of interest, and (iv) we could compute the mother's PoA when the child was 15–19. The latter allowed us to determine the distribution of the association between the study child's PoA at age 15–19 and nearly contemporary measures of her cognitive skills by level of the mother's PoA. Specifically, the negative association becomes stronger as contemporary PoA of the mother becomes faster.

The remainder of the paper is organized as follows. In Section 2 we describe the data; in Section 3 we present an overview of the models we estimate; Section 4 illustrates the main results; in Section 5 we perform some robustness check analyses; our findings are discussed in Section 6; Section 7 concludes the paper.

## 2 The data

The Avon Longitudinal Study of Parents and Children (ALSPAC), also known as Children of the 90s, is a British birth cohort study gathering clinic and questionnaire information on more than 14,500 families in the Bristol area. For more than two decades, detailed information has been collected on the children of these families, born between 1991 and early 1993 (see Boyd et al., 2013; Fraser et al., 2013*)*. The study website (http://www.bristol.ac.uk/alspac/researchers/our-data/) contains details of all the data that is available through a fully searchable data dictionary and variable search tool.^3^ The data includes, among others, linked administrative data on children's Key Stage performance from the National Pupil Database (NPD), a register of all pupils in public schools in England. The Key Stage defines any of the four fixed stages into which the English national scholastic curriculum is divided. At the end of each stage, children are required to complete standard final examinations. The average grades are obtained by averaging over these final examinations. For the children in our sample, Key Stage 1 (KS1) and Key Stage 4 (KS4) average grades were obtained at the age of 6–7 and at the age of 15–17, respectively.

DNA methylation data was collected from blood samples of around 1000 pairs of mother and child at three different points in time (see Relton et al., 2015): the child's birth (for both mothers and children), child age 7–9 (only for children) and child age 15–19 (for both mothers and children). The DunedinPoAm-measured pace of epigenetic aging was calculated for the children at all three time points according to Belsky et al. (2020). Consent for biological samples was collected in accordance with the Human Tissue Act (2004).

Finally, ALSPAC provides extensive information on children's family environment. Our main variable of interest is the parental social class, proxied by the paternal social class at birth.^4^ While social class is a complex concept that can be defined and measured in a variety of ways, occupation-based measures of social class (as the one we use here) have been shown to be useful and reliable tools that accurately capture socio-economic positioning (see, for reference, the review of Connelly et al., 2016). We here focus on paternal rather than maternal social class (observed during the pregnancy) for the following reasons: since social class is based on the latest occupation, women's social class during the pregnancy might already reflect changes in their occupation and career linked to the child penalty or its anticipation (see Goldin, 2014, and Goldin & Katz, 2016, for recent work on the subject). As men are less likely to experience career disruptions as a consequence of fatherhood, paternal social class might be a more appropriate measure of the socio-economic standing of the family a child is born into.^5^ In addition, paternal and maternal social class are highly correlated (ρ = 0.44) as one would expect from assortative matching patterns, with women being slightly more likely to match with men in higher social classes than their own. We here grouped paternal social class into three different categories: ‘professional’; ‘non-manual’ (which includes managerial and non-manual skilled fathers); ‘manual’ (which includes manual-skilled, partly-skilled and unskilled fathers).

We estimated the linear models described in Section 3 accounting for a rich set of controls, which we divided into five blocks. The first block includes base controls such as gender, being the first born, and the chronological age at which the KS1 and KS4 average grades were obtained. The second block includes variables observed during the pregnancy of the mother. The third block includes different dimensions of the socioeconomic status of the family. The fourth block includes characteristics of the child, such as body mass index (BMI), and whether the child smoked or drank at age 15. The fifth block includes a series of life events, observed from age 0 to age 11 of the child. A detailed description of the different blocks and the variables included in each of them can be found in Appendix A.

**Fig. 1:**
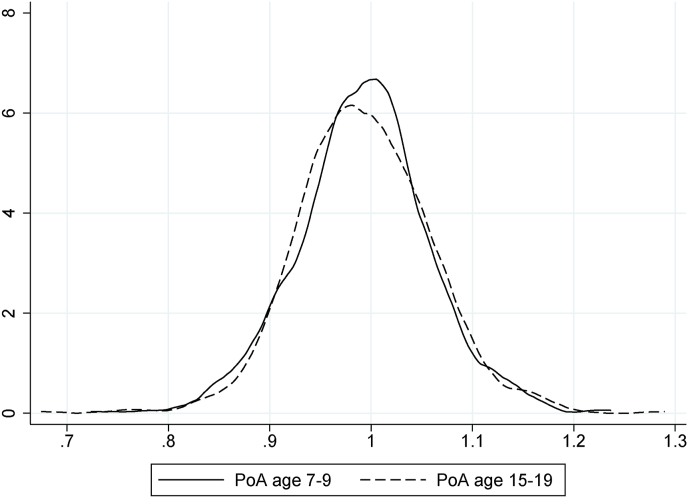
Kernel density PoA (ages 7–9 and 15–19) *Notes:* Distribution of pace of aging (ages 7–9 and 15–19) in sample.

Our sample was reduced to 700 units after cleaning of the methylation data and the exclusion of the children for whom either KS1 average grades or KS4 average grades or PoA at age 7–9 or PoA at age 15–19 were not observed.^6^ We followed Clark et al. (2021) for imputation of the missing data. When information on the count of mother's major financial problems (MFP, as defined in Appendix A) in some waves was missing, we replaced it by the mother's MFP count in the available waves, multiplied by the ratio of the total number of waves to the observed number of waves. When the information was not available in any wave, we replaced the missing value with the total sample mean and introduce a missing-value dummy as a right-hand side variable, as we did for the remaining family environment variables. The descriptive statistics of our final sample, including the percentage of imputed data per each variable, are reported in Table 1.^7^ PoA is expected to be normally distributed with mean equal to 1. Values lower and greater than 1 respectively indicate slower and faster PoA, as compared to the reference population. Fig. 1 reports the distribution of PoA at ages 7–9 and 15–19, both following a bell shape.

**Table 1:** Descriptive statistics main analysis.

	Type	Mean	Std. dev.	Min.	Max.	% Imputed
PoA (age 15–19)	Continuous	0.99	0.07	0.67	1.29	0
PoA (age 7–9)	Continuous	0.99	0.07	0.72	1.24	0
KS4 average grades	Continuous	463.47	119.47	34.5	887.25	0
KS1 average grades	Ordinal	10.82	3.21	1	15	0
**Base**						
Age PoA (age 15–19)	Continuous	17.08	1.05	15	19.25	0
Age PoA (age 7–9)	Continuous	7.45	0.13	7.25	8.42	0
Age KS4	Continuous	16.23	0.31	15.75	16.67	0
Age KS1	Continuous	7.23	0.31	6.75	7.67	0
First born	Binary	0.43	.	.	.	1.43
Female	Binary	0.51	.	.	.	0
**Pregnancy**						
Folic acid intake	Binary	0.08	.	.	.	0.86
Caesarean	Binary	0.08	.	.	.	4.14
Smoke in pregnancy	Binary	0.14	.	.	.	10.71
Heavy drink in pregnancy	Binary	0.03	.	.	.	7.29
Birth weight (kg)	Continuous	3.50	0.49	1.49	5.14	1.71
**Socioeconomic status**						
Age of the mother at birth	Continuous	29.51	4.54	18	42	0.43
Maternal education:						
CSE, vocational, O-level	Binary	0.54	.	.	.	1.86
A-level	Binary	0.27	.	.	.	1.86
Degree	Binary	0.17	.	.	.	1.86
Paternal social class:						
Manual	Binary	0.37	.	.	.	7.71
Non-manual	Binary	0.41	.	.	.	7.71
Professional	Binary	0.14	.	.	.	7.71
MFP count (age 0–7)	Ordinal	0.72	1.30	0	6	1.71
MFP count (age 0–11)	Ordinal	0.84	1.46	0	8	0.71
Average annual income FRS (age 0–7)	Continuous	21.28	9.49	3.21	37.04	5.29
Average annual income FRS (age 0–11)	Continuous	23.55	9.71	3.11	47.93	2.00
Log average annual income FRS (age 0–7)	Continuous	2.93	0.52	1.17	3.61	5.29
Log average annual income FRS (age 0–11)	Continuous	3.06	0.48	1.13	3.87	2.00
FDS (age 0–7)	Continuous	2.50	2.68	0	12.33	1.71
FDS (age 0–11)	Continuous	2.20	2.32	0	11.75	4.86
Mother has own house (age 7)	Binary	0.81	.	.	.	0.43
Mother has own house (age 11)	Binary	0.82	.	.	.	0
**Children**						
BMI (age 7)	Continuous	16.24	2.13	12.65	29.15	0.57
BMI (age 15)	Continuous	21.37	3.49	14.70	41.63	7.57
Smoker (age 15)	Binary	0.09	.	.	.	8.71
Drink alcohol (age 15)	Binary	0.45	.	.	.	9.00
**Life events**						
Mother divorced (age 0–6)	Binary	0.05	.	.	.	22.14
Mother divorced (age 0–11)	Binary	0.09	.	.	.	27.57
Mother or partner cruel to child (0–6)	Binary	0.12	.	.	.	22
Mother or partner cruel to child (0-11)	Binary	0.15	.	.	.	27.43
Diagnosed depression mother (age 6)	Binary	0.08	.	.	.	11.00
Diagnosed depression mother (age 12)	Binary	0.08	.	.	.	16.14
Mother has partner (age 6)	Binary	0.85	.	.	.	10.71
Mother has partner (age 12)	Binary	0.79	.	.	.	15.00
Any traumatic experience (age 0–7)	Binary	0.07	.	.	.	12.86
Any traumatic experience (age 0–10)	Binary	0.13	.	.	.	12.86

*Notes*: Descriptive statistics are reported for the main estimation sample (700 observations). A description of all variables can be found in Appendix A. Only means (that can be interpreted as percentages) are reported for binary variables, as SD, Min and Max hold no statistical meaning for these variables. Imputed observations for binary, ordinal and categorical variables form a ‘missing’ category (not reported); imputed observations for continuous variables are set to be equal to the sample mean of the variable. ‘KS1’ and ‘KS4’ refer, respectively, to Key Stage 1 and Key Stage 4 average grades. As defined in Appendix A, ‘MFP’ stands for ‘Major Financial Problem’, ‘FDS’ is the ‘Financial Difficulty Score’, ‘BMI’ is the ‘Body Mass Index’, and ‘FRS’ refers to the ‘Family Resources Survey’.

## 3 Empirical implementation

First, we tested the association between the study child's PoA and her family environment, focusing on paternal social class when the child was born, by employing linear regressions with PoA (at ages 7–9 and 15–19) as the dependent variable. In a second set of linear regressions, we tested the association between the child's measures of cognitive skills and her PoA (at ages 7–9 and 15–19), using KS1 and KS4 average grades as the dependent variables. The first general model we estimated is

(1) $${PoAm}_{i\left(t\right)}=\beta_{1}s_{i}^{n}+\beta_{2}s_{i}^{m}+x_{i\left(b\right)}^{\prime }\gamma +\varepsilon_{i}\text{,}$$

where ${PoAm}_{i\left(t\right)}$ is the PoA obtained from the blood sample of child i at age t, with $t=t_{1},t_{2}$, $t_{1}\in \left(7;9\right)$ and $t_{2}\in \left(15;19\right)$. Depending on t, two separate specifications of the general model (1) were defined, one for t being in the range 7–9 and the other for t being in the range 15–19. The variable $s_{i}$ is the parental social class of child i, proxied by the father's social class when the child was born, with $s_{i}^{n}$ representing the ‘non-manual’ category and $s_{i}^{m}$ representing the ‘manual’ category. The coefficients $\beta_{1}$ and $\beta_{2}$ are the main parameters of interest. The estimates of these parameters should be interpreted with respect to the omitted paternal social class ‘professional’. The vector $x_{i\left(b\right)}$ contains blocks of covariates that capture child characteristics and the family environment of child i. Following our estimation strategy, we sequentially included five blocks of covariates in the vector $x_{i\left(b\right)}$, where each block is defined in Appendix A (excluding, for ${t=t}_{1}$, all the variables that are observed after age $t_{1}$). In other words, with b=1,…,5, we obtained five nested specifications for $t=t_{1}$ and five nested specifications for $t=t_{2}$. In total we estimated ten models, five for t in the range 7–9 and five for t in the range 15–19. As we also observe secondary-school identifiers, we included school fixed-effects in the vector $x_{i\left(b\right)}$, for $t=t_{2}$ and b=1,…,5. This is important, as it allowed us to keep constant school characteristics and to partial out any systematic variation in the test-scores that was due to the quality of the school and peers.

Our second and third general models are

(2) $${KS1}_{i}=\rho_{1}{PoAm}_{i\left(t_{1}\right)}+z_{i\left(b\right)}^{\prime }\vartheta +\omega_{i}$$

(3) $${KS4}_{i}=\delta_{1}{PoAm}_{i\left(t_{2}\right)}+z_{i\left(b\right)}^{\prime }\mu +\varepsilon_{i},\left(3\right)$$

where ${KS1}_{i}$ and ${KS4}_{i}$ are the KS1 and KS4 average grades obtained by child i that are nearly contemporary with ${PoAm}_{i\left(t_{1}\right)}$ and ${PoAm}_{i\left(t_{2}\right)}$, respectively. In other words, we used (i) ${PoAm}_{i\left(t\right)}$ with $t=t_{1}$ when KS1 average grade was the dependent variable, and (ii) ${PoAm}_{i\left(t\right)}$ with $t=t_{2}$ when KS4 average grade was the dependent variable. The vector $z_{i\left(b\right)}$ contains blocks of covariates that capture child characteristics and the family environment of child i, where each block is defined in Appendix A.

As for model (1), we sequentially included in the vector $z_{i\left(b\right)}$ five blocks of variables such that we estimated five nested specification for model (2) and five nested specification for model (3). In model (2), we excluded from the vector $z_{i\left(b\right)}$ all the variables that were observed after age $t_{1}$. The vector $z_{i\left(b\right)}$ differs from $x_{i\left(b\right)}$ as, for b=3,…,5, $s_{i}^{n}$ and $s_{i}^{m}$ were also included in $z_{i\left(b\right)}$. As we also have secondary-school identifiers, in model (3) we included school fixed-effects in the vector $z_{i\left(b\right)}$, for b=1,…,5.

For each child i, KS1 and KS4 could be observed slightly before (and be not perfectly contemporary with) PoA at age $t=t_{1}$ and at age $t=t_{2}$, respectively. We tackled this issue by including the variable ‘age PoA’, as defined in Section 2, by way of a quadratic polynomial to control for non-linear trends that may interfere in the estimated relationship between the child's PoA and her average grades.

For the sake of comparability in the estimated coefficients, continuous variables were standardized in all regressions, including all dependent variables, excluding family income (at ages 0–7 and 7–11) as it is log-transformed in regressions, and excluding ‘age KS4’, ‘age KS1’ and ‘age PoA’ which are non-meaningful controls. Each model was estimated by Ordinary Least Squares (OLS), using White's robust standard errors.

## 4 Results

### 4.1 The determinants of PoA

**Fig. 2:**
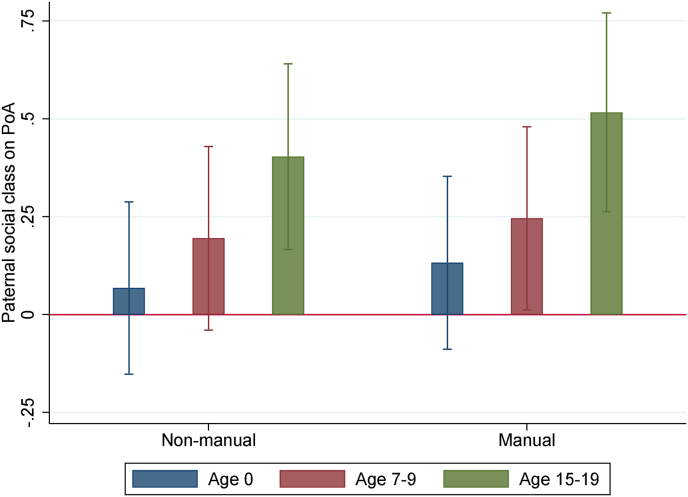
Paternal social class estimates on PoA at different ages: base specification *Notes:* The reference paternal social-class category is “professional”. Only base controls and, for age 15–19, school fixed-effects controls are included (see Section 3). For age 0 base controls include only ‘first born’, ‘female’ and ‘gestational age’ measured in weeks. Confidence intervals at the 95% level.

Fig. 2 displays, for b=1, the estimates of $\beta_{1}$ and $\beta_{2}$, the coefficients of the variables $s_{i}^{n}$ and $s_{i}^{m}$ in model (1), with omitted reference category ‘professional’ and with standardized dependent variable PoA at both ages. We also included the estimates of $\beta_{1}$ and $\beta_{2}$ for standardized PoA at age 0 (which is also available in our data), controlling for ‘First born’, ‘Female’ and gestational age measured in weeks.

**Fig. 3:**
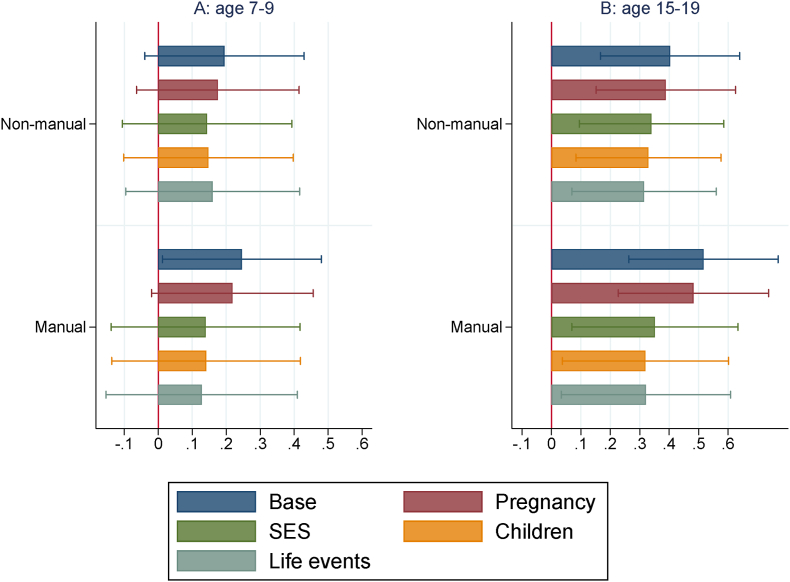
Paternal social class estimates on PoA at different ages: nested specifications *Notes:* The reference paternal social-class category is “professional”. Base specification includes, for age 15–19, school fixed-effects. Then, four blocks of controls are sequentially added: pregnancy, socioeconomic status (SES), children, life events (see Appendix A). Confidence intervals at the 95% level.

In Fig. 3 we plot the estimates of $\beta_{1}$ and $\beta_{2}$, for b=1,…,5, with standardized dependent variable PoA at both ages 7–9 and 15–19. Fig. 2, Fig. 3 are based on the regressions displayed in Table 2, Table 3.

**Table 2:** OLS estimates on PoA (age 7–9).

	model 1	model 2	model 3	model 4	model 5
Paternal social class:					
Non-manual	0.195	0.175	0.143	0.148	0.160
	(0.119)	(0.122)	(0.127)	(0.127)	(0.130)
Manual	0.246**	0.218*	0.139	0.141	0.128
	(0.119)	(0.121)	(0.141)	(0.141)	(0.143)
**Base**					
First born	0.086	0.097	0.123	0.121	0.171*
	(0.077)	(0.078)	(0.086)	(0.086)	(0.088)
Age PoA (age 7–9)	−10.737	−12.134	−13.044	−12.691	−13.536
	(8.845)	(8.838)	(9.502)	(9.569)	(9.722)
Age PoA squared (age 7–9)	0.703	0.794	0.853	0.829	0.881
	(0.571)	(0.570)	(0.616)	(0.620)	(0.630)
Female	0.018	0.018	0.010	0.010	0.010
	(0.076)	(0.077)	(0.077)	(0.077)	(0.077)
**Pregnancy**					
Folic acid intake in pregnancy		−0.214	−0.210	−0.205	−0.248*
		(0.141)	(0.142)	(0.142)	(0.137)
Caesarean		0.056	0.047	0.026	−0.066
		(0.124)	(0.126)	(0.130)	(0.134)
Smoke in pregnancy		0.129	0.077	0.072	0.046
		(0.116)	(0.116)	(0.117)	(0.117)
Heavy drink in pregnancy		−0.050	−0.046	−0.045	−0.028
		(0.222)	(0.214)	(0.216)	(0.212)
Birth weight		0.023	0.030	0.023	0.006
		(0.037)	(0.038)	(0.038)	(0.038)
**Socioeconomic status**					
Age of the mother at birth			0.011	0.012	0.026
			(0.042)	(0.042)	(0.043)
Maternal education:					
A-level			−0.013	−0.014	−0.027
			(0.095)	(0.095)	(0.100)
Degree			−0.020	−0.012	−0.030
			(0.132)	(0.132)	(0.134)
MFP count (age 0–7)			−0.017	−0.017	−0.022
			(0.045)	(0.045)	(0.046)
Log average annual income FRS (age 0–7)			−0.054	−0.062	−0.026
			(0.102)	(0.103)	(0.105)
FDS (age 0–7)			0.105**	0.106**	0.099**
			(0.050)	(0.050)	(0.050)
Mother has own house (age 7)			0.010	0.016	−0.007
			(0.121)	(0.121)	(0.123)
**Children**					
BMI (age 7)				0.032	0.047
				(0.045)	(0.045)
**Life events**					
Mother divorced (age 0–6)					0.240
					(0.174)
Mother or partner cruel to child (age 0–6)					0.278**
					(0.116)
Diagnosed depression mother (age 6)					−0.194
					(0.144)
Mother has partner (age 6)					0.072
					(0.221)
Any traumatic experience (age 0–7)					−0.080
					(0.140)
Observations	700	700	700	700	700
Adjusted R-squared	0.005	0.002	0.007	0.006	0.015

*Notes*: *p < 0.1**p < 0.05, ***p < 0.01. The outcome variable is standardized, as are the following controls: birth weight, age of the mother at birth, MFP count, FDS, and BMI. For missing continuous variables the mean is taken and then the missing value is controlled for. For categorical variables a category is added for missing values. Model 1: base controls; Model 2: base, pregnancy controls; Model 3: base, pregnancy, socioeconomic status controls; Model 4: base, pregnancy, socioeconomic status, children controls; Model 5: base, pregnancy, socioeconomic status, children, life events controls. Robust standard errors are reported in parentheses.

**Table 3:** OLS estimates on PoA (age 15–19).

	model 1	model 2	model 3	model 4	model 5
Paternal social class:					
Non-manual	0.403***	0.389***	0.340***	0.329***	0.314**
	(0.121)	(0.121)	(0.125)	(0.125)	(0.125)
Manual	0.516***	0.483***	0.351**	0.319**	0.321**
	(0.129)	(0.130)	(0.144)	(0.144)	(0.146)
**Base**					
First born	0.023	0.010	−0.096	−0.095	−0.109
	(0.083)	(0.086)	(0.091)	(0.090)	(0.090)
Age PoA (age 15–19)	−0.750	−0.283	−0.095	−0.098	0.299
	(1.738)	(1.724)	(1.758)	(1.815)	(1.903)
Age PoA squared (age 15–19)	0.025	0.011	0.006	0.007	−0.006
	(0.052)	(0.052)	(0.053)	(0.055)	(0.057)
Female	0.065	0.058	0.055	0.038	0.050
	(0.085)	(0.086)	(0.089)	(0.088)	(0.087)
**Pregnancy**					
Folic acid intake in pregnancy		−0.099	−0.083	−0.127	−0.075
		(0.168)	(0.163)	(0.163)	(0.170)
Caesarean		−0.061	−0.026	−0.085	−0.078
		(0.134)	(0.140)	(0.144)	(0.145)
Smoke in pregnancy		0.327**	0.263*	0.229*	0.234*
		(0.136)	(0.135)	(0.131)	(0.131)
Heavy drink in pregnancy		0.074	0.174	0.143	0.179
		(0.196)	(0.199)	(0.213)	(0.221)
Birth weight		−0.035	−0.044	−0.058	−0.064
		(0.041)	(0.041)	(0.041)	(0.041)
**Socioeconomic status**					
Age of the mother at birth			−0.164***	−0.165***	−0.169***
			(0.051)	(0.051)	(0.052)
Maternal education:					
A-level			−0.035	−0.019	−0.027
			(0.103)	(0.102)	(0.105)
Degree			−0.091	−0.060	−0.052
			(0.132)	(0.132)	(0.136)
MFP count (age 0–11)			−0.015	−0.012	0.001
			(0.047)	(0.046)	(0.044)
Log average annual income FRS (age 0–11)			−0.022	−0.047	−0.008
			(0.134)	(0.134)	(0.133)
FDS (age 0–11)			−0.001	−0.001	0.018
			(0.048)	(0.048)	(0.051)
Mother has own house (age 11)			−0.120	−0.097	−0.128
			(0.125)	(0.124)	(0.138)
**Children**					
BMI (age 15)				0.085*	0.083*
				(0.047)	(0.048)
Smoker (age 15)				0.432**	0.432**
				(0.179)	(0.183)
Drink alcohol (age 15)				−0.025	−0.047
				(0.092)	(0.094)
**Life events**					
Mother divorced (age 0–11)					0.056
					(0.166)
Mother or partner cruel to child (age 0–11)					−0.281**
					(0.140)
Diagnosed depression mother (age 12)					0.077
					(0.154)
Mother has partner (age 12)					−0.219
					(0.220)
Any traumatic experience (age 0–10)					−0.084
					(0.132)
Observations	700	700	700	700	700
Adjusted R-squared	0.028	0.030	0.039	0.055	0.063

*Notes*: *p < 0.1**p < 0.05, ***p < 0.01. The outcome variable is standardized, as are the following controls: birth weight, age of the mother at birth, MFP count, FDS, and BMI. For missing continuous variables the mean is taken and then the missing value is controlled for. For categorical variables a category is added for missing values. Model 1: base and school fixed-effects controls; Model 2: base, pregnancy and school fixed-effects controls; Model 3: base, pregnancy, socioeconomic status and school fixed-effects controls; Model 4: base, pregnancy, socioeconomic status, children and school fixed-effects controls; Model 5: base, pregnancy, socioeconomic status, children, life events and school fixed-effects controls. School fixed-effects are not reported. Robust standard errors are reported in parentheses.

Accounting for the base controls, we see from Fig. 2 that the paternal social class gradient in the child's PoA gradually emerges over time. While there are no significant differences in PoA at birth across social classes, children whose fathers belong to the lowest social class (‘manual’) have a faster PoA at age 7–9 as compared to children whose fathers belong to the highest social class (‘professional’). Fig. 3A shows that this holds true accounting for the base controls and, at the 90% level, for the pregnancy controls. This association is not robust to sequentially adding the other blocks of controls (socioeconomic status, children and life events). Fig. 2 additionally shows that, in adolescence, both children whose father belongs to the ‘manual’ and ‘non-manual’ social classes have a faster PoA, with respect to the children whose fathers belong to the ‘professional’ class. From Fig. 3B it is also evident that these associations are robust, at the 95% level, to sequentially adding all blocks of controls. These results suggest that having the father in lower social classes when the child is born is related to an increase in the study child's PoA and this association is stronger in adolescence than in childhood. By using maternal instead of paternal social class we obtain a slightly weaker association with PoA at age 15–19, which is not robust to all blocks of controls, implying that PoA in adolescence might be more sensitive to the paternal than the maternal social class at birth.^8^

A clearer insight into the estimates displayed in Fig. 2, Fig. 3 is given by the regression results in Table 2, Table 3 Few other variables seem to be significantly associated with the child's PoA at age 7–9 in Table 2. At the 95% level, these include the financial difficulties score (FDS) at age 0–7 (positive association) and mother or partner being cruel to the child at age 0–6 (positive association), suggesting that multidimensional poverty and childhood adversities may play a role in increasing the study child's PoA already at age 7–9.

As evident from Table 3, more variables manifest a consistent association with the child's PoA at age 15–19 over the blocks of controls. These include, at the 95% level, the age of the mother at birth, a proxy of the socioeconomic status of the family (Alessie et al., 2019), which is negatively associated with PoA at age 15–19, being a smoker at age 15 (positive association), and mother or partner being cruel to child at age 0–11, which surprisingly displays a negative association. At the 90% level, other variables that are somewhat consistently associated with the child's PoA at age 15–19 include BMI at age 15 (positive association) and having the mother who smoked in pregnancy (positive association).^9^ By comparing the estimates of the parameters of the categorical variables, it emerges that having the father in either the ‘manual’ or the ‘non-manual’ social class is associated with an increase of approximately 0.3–0.4 standard deviations in PoA at age 15–19, as compared to having the father in the ‘professional’ social class. The magnitude of this point estimate is comparable with that of being a smoker at age 15 and it is remarkably larger than that of the mother smoking in pregnancy.

### 4.2 Decomposition of the association between PoA and social class

To further investigate the contribution of each block of controls in shaping the association between paternal social class when the child is born and the child's PoA at age 15–19 we performed a mediation analysis as in Gelbach (2016). This method is based on the decomposition of the omitted-variable bias formula, derived from comparing the association of interest in the full model (b=5) and in the base model (b=1). The Gelbach decomposition is reported in Table 4. For simplicity, instead of categorizing paternal social class at birth in the three categories, we here grouped the ‘manual’ and ‘non-manual’ social classes together, to form a ‘low paternal social class’ indicator and thus be able to focus on one single association of interest (low paternal social class and PoA). In the ‘Base’ column we show the estimated association between the father being in the low social class and the child's PoA at age 15–19, accounting for the base controls and school fixed-effects (b=1). In the ‘Full’ column we report the estimated association between the father being in the low social class and the child's PoA at age 15–19, accounting for all the blocks of controls and school fixed-effects (b=5). The difference in the estimated association between ‘low paternal social class’ and PoA at age 15–19 between the ‘Full’ specification and the ‘Base’ specification (reported in the first row of column ‘Explained’), is then decomposed based on the contribution of the four blocks of controls (pregnancy, socioeconomic status, children and life events). The estimated contribution of each block is reported in the column ‘Explained’, starting from the second line. Since paternal social class was observed at birth of the study child (and was likely the same before the pregnancy started), the Gelbach decomposition should describe the mediating role of each block in the relation between paternal social class when the child was born and the child's PoA at age 15–19. According to the estimates, only the socioeconomic status block appears to have a (marginally) significant mediating role, probably driven by the negative association between age of the mother at birth and the child's PoA at age 15–19 (see Table 3). The mediating effect of age of the mother at birth seems to be small, as regressing the child's PoA at age 15–19 on the full battery of controls and on the same controls without the age of the mother at birth results in a minimal difference in the estimates of paternal social class (slightly higher point estimates in the second case). Moreover, only a marginally significant association between age of the mother at birth and the ‘manual’ social class was found by regressing age of the mother at birth on paternal social class and the full battery of controls.^10^ The strong association that we observe between paternal social class when the child was born and the child's PoA at age 15–19 and the Gelbach decomposition estimates thus suggest that there may be a direct causal effect of paternal social class at birth on PoA in adolescence.

**Table 4:** Gelbach decomposition based on paternal social class estimates on PoA (age 15–19).

	Base	Full	Explained
Low paternal social class	0.448***	0.316**	0.132**
	(0.113)	(0.122)	(0.059)
Pregnancy block			0.020
			(0.022)
Socioeconomic status block			0.086*
			(0.050)
Children block			0.017
			(0.019)
Life events block			0.010
			(0.024)

*Notes*: *p < 0.1**p < 0.05, ***p < 0.01. The outcome variable is standardized, as are the following controls: birth weight, age of the mother at birth, MFP count, FDS, and BMI. The base specification and the full specification columns report the estimated correlation between ‘low paternal social class’ (a dummy equal to 1 if the paternal social class at birth is ‘manual’ or ‘Non-manual’ and equal to 0 otherwise) and PoA (age 15–19), using base (including school fixed-effects) controls and the full battery of controls, respectively. The last column reports a decomposition of the difference between the estimated correlation in the base and in the full specifications, based on Gelbach (2016).

### 4.3 Faster PoA is associated with lower test scores in adolescence

**Fig. 4:**
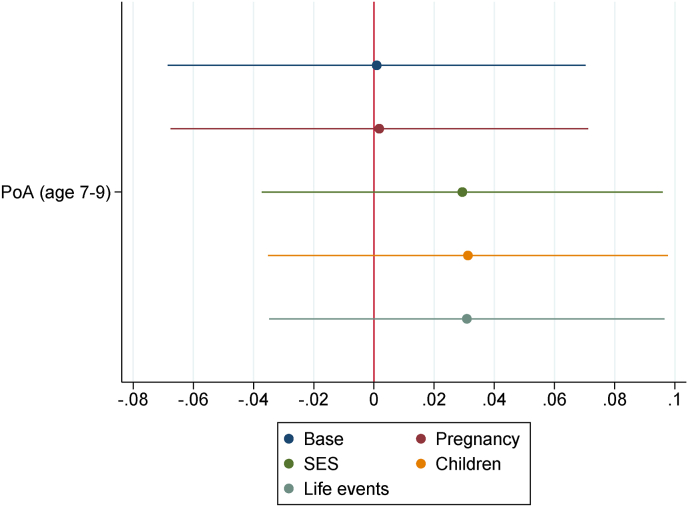
PoA (age 7–9) estimates on KS1 (age 6–7) *Notes:* To the Base specification four blocks of controls are sequentially added: pregnancy, socioeconomic status, children, life events (see Appendix A). Confidence intervals at the 95% level.

**Fig. 5:**
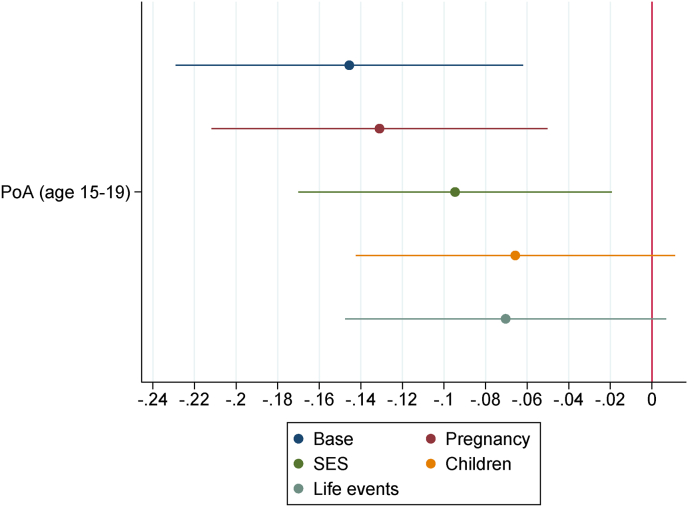
PoA (age 15–19) estimates on KS4 (age 15–17) *Notes:* Base specification includes school fixed-effects. Then, four blocks of controls are sequentially added: pregnancy, socioeconomic status, children, life events controls (see Appendix A). Confidence intervals at the 95% level.

Fig. 4, Fig. 5 display the estimates of $\rho_{1}$ and $\delta_{1}$, respectively. These coefficients are based on the estimates of models (2) and (3), where KS1 and KS4 are the main outcomes. Here the estimates of $\rho_{1}$ are virtually zero and never significant at conventional statistical levels, suggesting an absence of correlation between the child's PoA at age 7–9 and her contemporary measures of cognitive skills. However, the estimates of $\delta_{1}$ are significant at the 95% level when the first three blocks of controls are included (base, pregnancy and socioeconomic status controls), with the child's PoA at age 15–19 being negatively associated with her contemporary KS4 average grades. The association is significant at the 90% level only as the last two blocks of controls are included. Regression estimates of models (2) and (3) are reported in Appendix B Tables B1 and B2, respectively.

As mentioned above, based on Fig. 5, the estimated association between the child's PoA at age 15–19 and her KS4 average grades drops in magnitude over the sequentially added blocks of controls (while always remaining statistically different from zero at the 90% level). We performed a Gelbach decomposition to further investigate this result and detect which blocks of controls have a leading role in shaping this association. The decomposition is reported in Appendix B Table B3. Different from above, this decomposition should not be seen as a mediation analysis, but rather as a moderation analysis, as the child's PoA at age 15–19 could be itself a mediator of the relationship between the blocks of controls and the child's KS4 average grades. Moreover, there might be reverse causation between KS4 average grades, a cognitive skills measure, and PoA at age 15–19. Now around half of the child's KS4 average grades disparity, associated with faster PoA at age 15–19, is significantly explained by the socioeconomic status and the children blocks. This suggests a significant confounding effect of childhood socioeconomic status and children variables on the relationship between the child's PoA at age 15–19 and her KS4 average grades.

## 5 Sensitivity analyses

We performed a series of sensitivity tests. First, we used the IQ scores measured at the ages of 8 and 15 as the outcome variables in model (2) and (3), instead of nearly contemporary KS1 and KS4 average grades, to check for robustness in the results about the association between cognitive skills measures and PoA. IQ was the only other measure of cognitive abilities that was consistently available for children of ages 8 and 15 in our sample, but the validity of this measure is not undisputed. Both its ability to capture latent intelligence and its comparability across socio-economic, ethnic and cultural contexts has been widely debated (see Shuttleworth-Edwards et al., 2004, for a review of some of the recent literature). However, given the relative homogeneity of our ALSPAC subsample both in terms of ethnicity (all children in our sample are Caucasian) and socio-economic status (Avon is a comparatively rich county in the UK), we are not particularly worried about ethnic background or a middle-class bias playing a major role in the association between social class and IQ.^11^ Point estimates from these secondary analyses should nevertheless be interpreted with caution; we here take them mostly as a test of convergent validity for our preferred measures of cognitive skills (KS1 and KS4 average grades). These secondary analyses were performed using only those children, among the 700 in our main sample, for whom IQ scores were available in both periods (606 children). Descriptive statistics of the IQ scores at the ages of 8 and 15 and of the blocks of control variables in this subsample are reported in Table B4 in Appendix B. In Figures B1 and B2 in Appendix B we plot the estimates of $\rho_{1}$ and $\delta_{1}$, still based on models (2) and (3), but where the dependent variable is now the child's IQ scores at the ages of 8 and 15 instead of her KS1 and KS4 average grades. Figures B1 and B2 are based on Tables B5 and B6 in Appendix B, respectively. It can be seen that using the IQ scores at the age of 8 leads to very similar results (non-significant association between IQ scores at age 8 and PoA at age 7–9) compared to using KS1 average grades as the dependent variable. Using IQ scores at the age of 15 instead of KS4 average grades leads to somewhat similar results, although the estimated association with PoA at age 15–19 seems to be weaker. For b=1,2 this association is still significant at the 95% level, although it becomes non-significant from b=3 onward, that is once we introduce the socio-economic block of controls. Variables that are significantly associated with the child's IQ at age 15 at the 95% level include female (negative association), maternal education (higher maternal education associated with higher IQ scores), paternal social class (‘manual’ social class associated with lower IQ scores), being a smoker at age 15 (negative association), mother has a partner when the child is 12 (negative association) and any traumatic experience at age 0–7 (negative association). Similar to what shown in Appendix B Table B3 for the KS4 average grades, the Gelbach decomposition reported in Table B7 in Appendix B shows that the socioeconomic status block of controls significantly shapes the association between the child's IQ scores at age 15 and her PoA at age 15–19.

Second, we augmented the regressions reported in Appendix B Tables B2 and B6 by adding an interaction term between the child's standardized PoA at age 15–19 and the contemporary (standardized) PoA of her mother. The negative association between the child's cognitive outcomes and accelerated PoA in adolescence might be mitigated or enhanced by her mother's own PoA. Keeping age and socio-economic characteristics constant, mothers with a slower PoA might be better able to provide their children with the material investments and nurturing environment that would foster the child's cognitive abilities, thus counterbalancing the adverse cognitive effects of the child's own faster PoA. For the regression on standardized KS4 average grades we used all the 700 units used in the main sample excluding the 34 children for whom the contemporary PoA of the mother was not available. For the regression on standardized IQ scores at age 15 we used all the 606 units used in the sample excluding the 29 children for whom contemporary PoA of the mother was not available. The estimated marginal effects are reported in Appendix B Tables B8 and B9, respectively, and are based on the regressions reported in Appendix B Tables B10 and B11, respectively. From level 0 (average value) to level 3 of PoA of the mother, the negative association between the child's PoA at age 15–19 and her cognitive skills measures becomes up to three times stronger and is generally robust to all our controls. This has some possible explanations that we investigate in Section 4.

Third, we estimated model (2) using KS2 and KS3 instead of KS1 average grades as the dependent variable. For these estimations we used those children, among the 700 in our main sample, for whom both KS2 and KS3 average grades are available in ALSPAC (479 children). KS2 and KS3 exit exams took place after PoA at age 7–9 was observed: KS2 average grades were obtained at child age 10–11 and KS3 average grades at child age 13–14. The results (available upon request) are similar to the ones reported in Appendix Table B1, with PoA not being significantly associated with KS2 and KS3 average grades for b=1,…,5.

Fourth, we estimated model (1) and model (3) without imputation of the missing data on the control variables. As expected, the number of observations drops remarkably as does the power of the statistical test for joint significance. The estimated association between paternal social class and PoA at age 15–19 is still significant, with lower parental social classes associated with faster PoA, while the estimated negative association between PoA at age 15–19 and KS4 average grades becomes not significant, at the 90% level, after adding the socioeconomic status block of controls. These results are available upon request.

## 6 Discussion

In this paper we showed several results. First, we observed a strong association between the study child's paternal social class when the child is born and her PoA at age 15–19, with lower social classes associated with faster PoA. This association is robust to all blocks of controls that we sequentially included in the regressions, namely base, pregnancy, socioeconomic status, children and life events variables. We did not observe a similar strong association between the child's paternal social class at birth of the child and her PoA at age 7–9 (and at age 0), which suggests that the paternal social class gradient in PoA might start appearing only later in adolescence. We do not find evidence of this result being driven by an ‘epigenetic drift’, that is the increased in epigenetic variability with age: the distribution of PoA over time is remarkably stable in our sample (as shown in Fig. 1). We observed that multidimensional poverty (larger FDS) and an emotionally or physically abusive mother or mother's partner, both from birth to age 6–7, are positively associated with faster PoA at age 7–9. We also observed that the age of the mother at birth, a proxy of the socioeconomic status of the family, is consistently associated with the child's PoA at age 15–19 (negative association) as well as being a smoker at age 15 (positive association).^12^ To summarize, it appears that the family socioeconomic status might play a role in affecting the child's PoA in the period from childhood to adolescence, as confirmed by the Gelbach decomposition results. This is somewhat in line with the findings of Raffington et al. (2021a) and Raffington et al. (2021b) and is consistent with qualitative evidence from the review of Yoeli et al. (2021).

Second, we observed that the child's PoA at age 15–19 is negatively associated with her KS4 average grades obtained at the age of 15–17. This association is robust to all our blocks of controls, at least at the 90% level, although it drops remarkably in size as more blocks of controls are added. We did not observe any association of the child's PoA at age 7–9 with her KS1 average grades, obtained at the age of 6–7. In the robustness checks we used IQ scores observed at the age 8 and 15, instead of KS1 and KS4 average grades, and we estimated somewhat similar (albeit weaker) associations. The Gelbach decomposition suggests that the socioeconomic status block of variables plays a primary role in shaping the association between the child's PoA at age 15–19 and her KS4 average grades as well as her IQ scores at age 15.

By interacting the child's PoA at 15–19 and the contemporary PoA of her mother, we were able to estimate that the negative association between the child's PoA at 15–19 and her contemporaneous measures of cognitive skills (KS4 average grades and IQ scores at age 15) becomes stronger, as the level of PoA of her mother increases above the average. In other words, for the subpopulation of children whose mothers have a remarkably fast PoA, the negative association between the child's PoA and her cognitive skills measures is particularly strong and generally robust to all our blocks of controls.

While we did not find evidence of any significant association between PoA and cognitive skills measures in primary aged children, Ruffington et al. (2021b) show a negative correlation between PoA and scores in some cognitive tests (verbal comprehension and perceptual reasoning) in children and adolescents.^13^ Several reasons might explain the difference in the findings. First, their results might be driven by the association of PoA and scores in cognitive tests in adolescence only, as they used cross-sectional data on a mixed sample of children and adolescents while we exploited repeated measures of PoA observed at two points in time for the same individuals (i.e., in their childhood and in their adolescence); second, we used blood instead of salivary DNA methylation data, improving on the reliability of the results as the DunedinPoAm algorithm was developed on blood samples (Belsky et al., 2020); third, we used different measures of cognitive skills, providing evidence of convergent validity.

From our results, some implications for future research arise. The understanding of the mechanisms behind the paternal social class gradient in PoA seems to be of primary importance. Through the current work, we offer evidence that suggests the presence of a causal and direct effect of paternal social class on the PoA in adolescence. Our interpretation is supported by (i) the persistence of the association between the child's paternal social class at birth and her PoA at age 15–19 after the sequential inclusion of a wide range of controls, and (ii) the Gelbach decomposition, which suggests that only the socioeconomic status blocks of controls has a (marginally) significant role in mediating this association, probably driven by the age of the mother at birth whose mediating role appears to be small. It remains to understand whether PoA in adolescence acts as a mediator between socioeconomic status in childhood (or, more specifically, paternal social class at birth) and future health outcomes. If it does, PoA (in adolescence) might become an inexpensive diagnostic tool that can be simply calculated from blood samples. This tool can then be used to evaluate the effectiveness of policies targeting the negative health effects of low socioeconomic status in childhood (Raffington et al., 2021a). None of the few existing studies on the relationship between childhood socioeconomic status and PoA have claimed causality in their results, which might represent the *condicio sine qua non* of PoA becoming such a diagnostic tool. Consequently, more research is needed to disentangle the causal effect of PoA in adolescence on health later in life.

With respect to the second part of our paper, we provided evidence of a correlation between the child's PoA and her cognitive skills measures in adolescence. This correlation becomes weaker as more controls are included in our regressions. Based on our estimates and on the Gelbach decomposition, around half of the KS4 average grades disparity associated with a faster PoA at age 15–19 is accounted for by the socioeconomic status and the child control blocks. This result might be driven by the moderating effect of paternal social class at birth and smoking behaviour at age 15, which, in our regressions, were significantly associated with both the child's PoA at age 15–19 and her cognitive skills measures in adolescence. These results might point toward the absence of a causal relationship between the child's PoA at age 15–19 and her cognitive skills measures. This conclusion is strengthened by our estimates of the association between the child's PoA at age 15–19 and her IQ scores at age 15, which is even weaker. However, as mentioned above, the negative association between PoA at age 15–19 and contemporary measures of cognitive skills becomes stronger for the subpopulation of children whose mothers have PoA above the average level. This result opens up reflections on why, how and when parental resources are important for child development (Björklund & Salvanes, 2011). More specifically, it suggests that the intergenerational transmission of health and lifestyle through faster maternal PoA can lead to a stronger negative association between child's PoA and her cognitive skills measures.

All in all, the true causal effect might be difficult to identify in future research, as it might be hard to rule out reverse causation between PoA and cognitive skills measures. The solution to the issue could be using data on PoA observed at the early stages of adolescence, when children have no to little decisional power on making their own choices, and implementing twin fixed-effect designs. Then PoA at early-life stages would be randomly allocated between twins and, if enough variation can be exploited, its causal effect on cognitive skills measures over the life span can be identified. This method would rely on the assumptions that (i) identical twins share the same genes, pregnancy conditions, parental background and experiences in childhood, and (ii) twins represent the general population well. Alternatively, siblings fixed-effect designs might offer an approximation of the causal effect, when a battery of meaningful controls is also available, with the advantage of typically larger datasets and higher representativeness of the entire population. Whether cognitive skills measures are causally related to PoA is a relevant question. The link between childhood cognitive skills measures and health later in life, including, among others, mortality (e.g., Whalley and Deary, 2001) and mental health (e.g., Russ et al., 2017), is well established. The existence of a strong causal effect of PoA early in life on cognitive skills measures later in life would imply that individuals with a particularly fast PoA should be provided with tailored scholastic support aimed to increase school performance and boost cognitive abilities and, ultimately, have better (health) outcomes over the life span. As we identify a strong moderating effect of socioeconomic status (through paternal social class) on the association between the child's PoA and her cognitive skills measures in adolescence, future research should investigate whether PoA early in life acts itself as a mediator between socioeconomic status and cognitive ability. Then, PoA might become a diagnostic tool to detect whether policies aimed to boost cognitive abilities by tackling consequences of socioeconomic disadvantage are effective.

Our results do not imply that aging is somewhat pre-determined by our epigenome, nor that epigenetic processes are pre-ordained by social class. There is still much of the relationship between child PoA and parental social class that is unaccounted for in our models. In addition, our results are silent on the effectiveness that public policy interventions would have on PoA or whether we should expect treatment effects of such interventions to vary by social class. Nevertheless, our results can be useful to identify vulnerable groups that should be object of future policy. While the finding that people from lower social class are more at risk of epigenetic aging is not completely novel per se, the identification of the *timing* at which this risk emerges is. So overall, our results suggest that childhood is the critical period where public policy interventions could contrast the emergence of the PoA gradient, which starts to appear around 7–9 years of age and consolidates in adolescence. Last, epigenetic modifications can be shaped by the environment; so, even in cases where differences in PoA are already in place, changes in lifestyle have been shown to be effective in lowering epigenetic biomarkers of aging (see, for example, the diet and physical activity intervention trial in Fiorito et al., 2021). Thus, policies encouraging changes in lifestyle (especially for those from lower social classes) could help closing the social-class gap in PoA, with beneficial spillover effects on a variety of other outcomes (as hinted by our results on school grades).

## 7 Conclusion

In this paper we investigated the association between study children's PoA observed at two points in time (childhood and adolescence), their early-life environment and their measures of cognitive skills. We found that children with lower paternal social class when they are born display faster PoA at age 15–19 and that the paternal social class gradient in PoA emerges gradually from childhood to adolescence. Controlling for our blocks of controls (base, pregnancy, socioeconomic status, children and life events variables) did not account for this social class disparity in PoA. Moreover, only the socioeconomic status block of controls seems to have a (marginally) significant role in mediating the association between the child's paternal social class and her PoA at age 15–19. This suggests that there may be a direct causal effect of paternal social class when the child is born on the child's PoA in adolescence, although more work would be needed to understand the drivers of this dynamic effect. We also showed a negative correlation between the child's PoA at age 15–19 and her nearly contemporary cognitive skills measures. This correlation became weaker as more controls were added, suggesting that our controls contribute shaping this association. Around half of the KS4 average grades disparity, associated with faster PoA at age 15–19, was accounted for by the socioeconomic status and the children blocks, including variables such as paternal social class and smoking behavior at age 15. Similar results were obtained by estimating the association between the child's PoA at age 15–19 and her IQ scores at age 15, with a large part of the IQ scores disparity associated with faster PoA at age 15–19 accounted for by the socioeconomic status block of controls. Our results on the association between PoA in adolescence and cognitive skills measures seem to point towards the absence of a causal effect, although for the subpopulation of children whose mothers have a PoA above the average level, the negative association between PoA at 15–19 and cognitive skills measures in adolescence is up to three times stronger and generally robust to all our controls. Overall, our results seem to identify adolescence as the period of life when PoA, family environment and cognitive skills measures begin to interact.

## Ethical Statement for Solid State Ionics

Hereby, I Giorgia Menta consciously assure that for the manuscript “Pace of aging, family environment and cognitive skills in children and adolescents” the following is fulfilled:

1) This material is the authors' own original work, which has not been previously published elsewhere.
2) The paper is not currently being considered for publication elsewhere.
3) The paper reflects the authors' own research and analysis in a truthful and complete manner.
4) The paper properly credits the meaningful contributions of co-authors and co-researchers.
5) The results are appropriately placed in the context of prior and existing research.
6) All sources used are properly disclosed (correct citation). Literally copying of text must be indicated as such by using quotation marks and giving proper reference.
7) All authors have been personally and actively involved in substantial work leading to the paper, and will take public responsibility for its content.

The violation of the Ethical Statement rules may result in severe consequences.

To verify originality, your article may be checked by the originality detection software iThenticate. See also http://www.elsevier.com/editors/plagdetect.

I agree with the above statements and declare that this submission follows the policies of Solid State Ionics as outlined in the Guide for Authors and in the Ethical Statement.

## Author statement

Gianmaria Niccodemi: Formal analysis, Software, Writing – Original Draft. Giorgia Menta: Software, Data curation, Methodology, Writing – Review and editing. Conchita D'Ambrosio: Conceptualization, Resources, Supervision, Funding acquisition. Jonathan Turner: Supervision, Funding acquisition.

## Data Availability

The authors do not have permission to share data.

## Footnotes

1 Namely, HbA1C; Cardiorespiratory fitness; Waist-hip ratio; FEV1/FVC; FEV1; Mean arterial pressure; BMI; Leukocyte telomere length; Creatinine clearance; Urea Nitrogen; Lipoprotein; Triglycerides; Gum health; Total cholesterol; White blood cell count; hsCRP; HDL cholesterol; ApoB100/ApoA1.

2 As opposed to other epigenetic clocks, which are based on the cross-sectional comparison of younger and older individuals, PoA is trained on longitudinal data from individuals who are all the same chronological age, similar to ALSPAC participants. Additionally, even if it was trained on individuals aged 26, 32, and 38 years old, PoA has been shown to work well also in children and adolescents. (e.g., Belsky et al., 2020; Etzel et al., 2022; Raffington et al., 2021a; 2021b), providing results that are consistent with established findings in the literature on age acceleration in children.

3 Ethical approval for the study was obtained from the ALSPAC Ethics and Law Committee and the Local Research Ethics Committees. Informed consent for the use of data collected via questionnaires and clinics was obtained from participants following the recommendations of the ALSPAC Ethics and Law Committee at the time.

4 Social class in ALSPAC is derived from the 1991 occupational classification of the Office of Population Censuses and Surveys (OPCS, now National Statistics, NS).

5 We nevertheless check whether our results are sensitive to this particular choice, by using maternal social class in robustness checks.

6 All children in the estimation sample are of Caucasian ethnicity. This attenuates concerns linked to population stratification that can arise when comparing epigenetic outcomes of ethnically diverse populations (Barfield et al., 2014).

7 From Table 1, the life-event control block displays relatively higher rates of imputation as compared to other controls. This is likely due to the sensitivity of some of the items included in the block, which commonly have high degree of missingness in survey data. Regardless, the life event controls have a high mediating potential in the relationship between parental social class and child PoA, as they are likely linked to the former via shared environment and to the latter through epigenetic modifications that traumatic events can trigger.

8 Results available upon request.

9 As smoking tends to be associated with some of the biomarkers of organ system integrity on which the PoA algorithm was trained (e.g. cardiorespiratory fitness; FEV1), we are not surprised to see that the child smoking at age 15 is strongly correlated with her PoA around the same age. Similarly, maternal smoking during pregnancy could have both a direct (biological) and an indirect (behavioural) effect on the child's PoA. Given that the association between maternal smoking in pregnancy and child PoA is stronger at age 15–19 than it is earlier in childhood, our results seem to point to an indirect effect of maternal smoking on child PoA, transiting through behavioural channels that become more relevant as the child reaches adolescence.

10 Results available upon request.

11 We additionally test, on the larger sample of all ALSPAC participants, whether there exist any significant differences in IQ scores between Caucasian children and children from other ethnic backgrounds. T-tests of equality of means suggest that such differences are small and not statistically different from zero at conventional statistical thresholds: differences (p-values) are, respectively, 1.5 (0.32) and 2.2 (0.12) for IQ at age 8 and age 15.

12 As smoking is one of the 18 biomarkers of organ system integrity on which the PoA algorithm was trained, we are not surprised to see that smoking at age 15 is strongly correlated with the child's PoA around the same age. Similarly, maternal smoking during pregnancy could have both a direct (biological) and an indirect (behavioural) effect on the child's PoA. Given that the association between maternal smoking in pregnancy and child PoA is stronger at age 15–19 than it is earlier in childhood, our results seem to point to an indirect effect of maternal smoking on child PoA, that transits trhough behavioural channels that become more relevant as the child reaches adolescence.

13 Although they do not find any association between pace of aging and test scores on processing speed, executive functions, reading and math.
