# Supplementary material for: Pace of aging, family environment and cognitive skills in children and adolescents
Source: SSM Popul Health. 2022 Nov 5;20:101280. doi: 10.1016/j.ssmph.2022.101280 (PMC9661391; doi:10.1016/j.ssmph.2022.101280)
Supplement: Multimedia component 1 [file mmc1.pdf]

APPENDIX A: CONTROL VARIABLES

We here list and describe all the variables included in each block of controls:

1. **Base variables.** 'Female', a dummy equal to 1 if the child is a female and equal to 0 otherwise; 'First born', a dummy equal to 1 if the child is the first born of the family and equal to 0 otherwise; 'Age KS1 /KS4', the age at which KS average grades (KS1 or KS4, depending on the model) were obtained; 'Age pace of aging', the chronological age at which pace of aging was obtained through blood DNA methylation samples.

2. **Pregnancy variables.** 'Folic acid intake in pregnancy', a dummy equal to 1 if the mother took folic acid supplements during pregnancy and equal to 0 otherwise; 'Caesarean', a dummy equal to 1 if the child was born through caesarean section and equal to 0 otherwise; 'Smoke in pregnancy', a dummy equal to 1 if the mother ever smoked during pregnancy and equal to 0 otherwise; 'Heavy drink in pregnancy', a dummy equal to 1 if the mother drank at least 1-2 glasses of alcohol per day during the first three months of the pregnancy, or since the first move of the foetus, or during the last two months of the pregnancy; 'Birth weight'.

3. **Socioeconomic status variables.** 'Education of the mother', defined as the education of the mother at birth in 3 different categories: 'CSE, vocational or O-level'; 'A-level'; 'Degree'; 'MFP count' (ages 0 to 7 and 0 to 11), the number of major financial problems the mother encountered over the indicated age-range of the child; 'average annual income FRS' (ages 0 to 7 and 0 to 11), obtained by matching weekly income bands in our data with figures using data from the Family Resources Survey on the distribution of net household income in the South West region, deflated to 2008 prices (following Clark *et al*., 2021, with the same ALSPAC data); 'FDS score' (ages 0 to 7 and 0 to 11), a financial difficulties score constructed as the mean, across the waves, of the sum of different items of which difficulty in affordability was measured in the range 0-15, including food, heating, rent, clothing and child things; 'Mother has own house' (ages 7 and 11), a dummy variable equal to 1 if the mother owned a house at

the indicated child age. These variables capture the permanent income of the household, its wealth as proxied by home-ownership, multidimensional poverty via the FDS score and also the mother's perception of adequacy of available resources with the MFP indicator. All these dimensions of socio-economic status are necessary to have a complete picture of material well-being.

4. **Children variables.** 'BMI' (ages 7 and 15); 'Smoker' (age 15), a dummy equal to 1 if the child smoked at least 1 cigarette per week at age 15 and equal to 0 otherwise; 'Drink alcohol' (age 15), a dummy equal to 1 if the child experienced drinking 5 or more drinks over 24 hours in the previous 2 years and equal to 0 otherwise.

5. **Life events variables.** 'Mother divorced' (ages 0 to 6 and 0 to 11), a dummy equal to 1 if the mother divorced at least once over the indicated age-range of the child and equal to 0 otherwise; 'Mother or partner cruel to child' (ages 0 to 6 and 0 to 11), a dummy equal to 1 if the mother and/or the partner have ever been emotionally or physically abusive toward the child over the indicated age-range and equal to 0 otherwise; 'Diagnosed depression mother' (ages 6 and 12), a dummy equal to 1 if the mother was diagnosed with depression at the indicated child age; 'Any traumatic experience' (ages 0 to 7 and 0 to 10), a dummy equal to 1 if the child experienced his or her most traumatic event, if any, over the indicated age-range and equal to 0 otherwise.

APPENDIX B: ADDITIONAL FIGURES AND TABLES

Figure B.1: PoA (age 7-9) estimates on IQ (age 8)

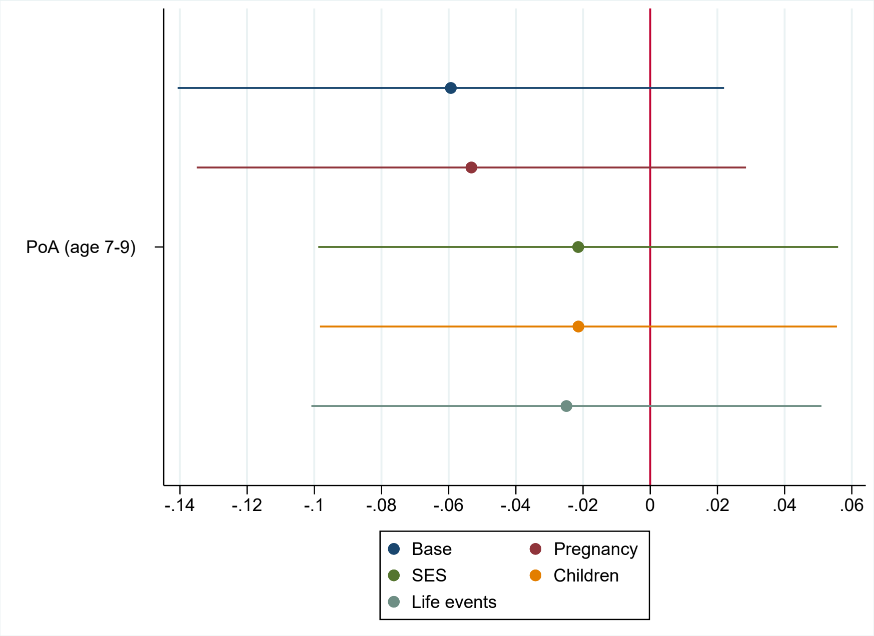

Notes: To the Base specification four blocks of controls are sequentially added: pregnancy, socioeconomic status (SES), children, life events (see Appendix A). Confidence intervals at the 95% level.

Figure B.2: PoA (age 15-19) estimates on IQ (age 15)

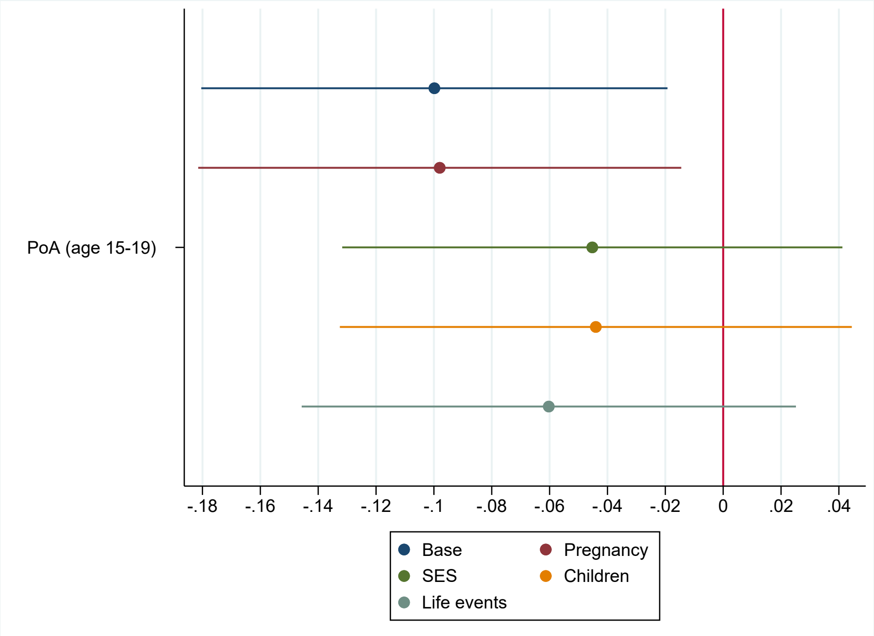

Notes: Base specification includes school fixed-effects. Then, four blocks of controls are sequentially added: pregnancy, socioeconomic status (SES), children, life events (see Appendix A). Confidence intervals at the 95% level.

Table B.1: OLS estimates on KS1 (age 6-7)

	model 1	model 2	model 3	model 4	model 5
PoA (age 7-9)	0.001	0.002	0.029	0.031	0.031
	(0.035)	(0.035)	(0.034)	(0.034)	(0.033)
**Base**					
First born	0.018	0.085	0.050	0.051	0.032
	(0.075)	(0.076)	(0.079)	(0.079)	(0.079)
Age KS1	0.722***	0.702***	0.710***	0.711***	0.728***
	(0.117)	(0.117)	(0.119)	(0.118)	(0.118)
Age PoA (age 7-9)	-5.526	-5.285	-6.280	-5.941	-5.948
	(14.039)	(13.921)	(14.930)	(14.400)	(14.578)
Age PoA squared (age 7-9)	0.306	0.296	0.376	0.358	0.364
	(0.926)	(0.918)	(0.986)	(0.951)	(0.963)
Female	0.213***	0.228***	0.223***	0.232***	0.229***
	(0.074)	(0.073)	(0.071)	(0.071)	(0.070)
**Pregnancy**					
Folic acid intake in pregnancy		0.000	-0.035	-0.036	-0.011
		(0.125)	(0.122)	(0.122)	(0.121)
Caesarean		-0.115	-0.092	-0.031	-0.071
		(0.141)	(0.133)	(0.137)	(0.132)
Smoke in pregnancy		-0.043	0.169	0.184	0.184
		(0.109)	(0.113)	(0.114)	(0.113)
Heavy drink in pregnancy		0.327*	0.184	0.190	0.177
		(0.193)	(0.189)	(0.187)	(0.185)
Birth weight		0.109***	0.098***	0.115***	0.102***
		(0.038)	(0.036)	(0.037)	(0.037)
**Socioeconomic status**					
Age of the mother at birth			-0.018	-0.022	-0.028
			(0.045)	(0.045)	(0.045)
Maternal education:					
A-level			0.252***	0.249***	0.232***
			(0.086)	(0.086)	(0.087)
Degree			0.352***	0.338***	0.334***
			(0.113)	(0.112)	(0.113)
Paternal social class:					
Non-manual			-0.273***	-0.265***	-0.270***
			(0.101)	(0.100)	(0.099)
Manual			-0.396***	-0.387***	-0.370***
			(0.115)	(0.114)	(0.112)
MFP count (age 0-7)			0.025	0.026	0.034
			(0.041)	(0.041)	(0.040)
Log average annual income FRS (age 0-7)			0.153	0.159*	0.205**
			(0.094)	(0.094)	(0.092)
FDS (age 0-7)			-0.002	-0.004	0.014
			(0.047)	(0.047)	(0.048)
Mother has own house (age 7)			0.146	0.137	0.026
			(0.117)	(0.117)	(0.119)
**Children**					
BMI (age 7)				-0.082**	-0.068*
				(0.037)	(0.037)
**Life events**					
Mother divorced (age 0-6)					-0.251
					(0.187)
Mother or partner cruel to child (age 0-6)					-0.176
					(0.118)
Diagnosed depression mother (age 6)					-0.210
					(0.131)
Mother has partner (age 6)					-0.359*
					(0.187)
Any traumatic experience (age 0-7)					-0.245
					(0.153)
Observations	700	700	700	700	700
Adjusted R-squared	0.070	0.086	0.156	0.160	0.177

Notes: $^{*}p < 0.1$ $^{**}p < 0.05$, $^{***}p < 0.01$. The outcome variable is standardized, as are PoA and the following controls: birth weight, age of the mother at birth, MFP count, FDS, and BMI. For missing continuous variables the mean is taken and then the missing value is controlled for. For categorical variables a category is added for missing values. Model 1: base controls; Model 2: base, pregnancy controls; Model 3: base, pregnancy, socioeconomic status controls; Model 4: base, pregnancy, socioeconomic status, children controls; Model 5: base, pregnancy, socioeconomic status, children, life events controls. Robust standard errors are reported in parentheses.

Table B.2: OLS estimates on KS4 (age 15-17)

	model 1	model 2	model 3	model 4	model 5
PoA (age 15-19)	-0.146***	-0.131***	-0.095**	-0.066*	-0.070*
	(0.043)	(0.041)	(0.038)	(0.039)	(0.039)
**Base**					
First born	0.129*	0.137*	0.125	0.112	0.108
	(0.078)	(0.078)	(0.081)	(0.079)	(0.080)
Age KS4	0.125	0.119	0.161	0.155	0.115
	(0.129)	(0.130)	(0.127)	(0.126)	(0.127)
Age PoA (age 15-19)	-0.094	-0.389	0.123	-0.158	-0.318
	(1.664)	(1.662)	(1.536)	(1.543)	(1.549)
Age PoA squared (age 15-19)	0.006	0.014	-0.002	0.006	0.011
	(0.050)	(0.050)	(0.046)	(0.046)	(0.046)
Female	0.226***	0.216***	0.223***	0.233***	0.234***
	(0.081)	(0.080)	(0.076)	(0.075)	(0.075)
**Pregnancy**					
Folic acid intake in pregnancy		-0.128	-0.100	-0.051	-0.015
		(0.135)	(0.125)	(0.123)	(0.123)
Caesarean		0.105	0.139	0.180	0.197
		(0.154)	(0.143)	(0.143)	(0.142)
Smoke in pregnancy		-0.354***	-0.150	-0.113	-0.119
		(0.125)	(0.123)	(0.122)	(0.121)
Heavy drink in pregnancy		-0.129	-0.214	-0.192	-0.200
		(0.194)	(0.204)	(0.203)	(0.204)
Birth weight		0.013	0.013	0.036	0.037
		(0.041)	(0.039)	(0.038)	(0.039)
**Socioeconomic status**					
Age of the mother at birth			0.007	0.011	0.004
			(0.045)	(0.044)	(0.044)
Maternal education:					
A-level			0.234**	0.212**	0.224**
			(0.094)	(0.093)	(0.094)
Degree			0.485***	0.462***	0.492***
			(0.129)	(0.129)	(0.130)
Paternal social class:					
Non-manual			-0.003	-0.014	0.016
			(0.109)	(0.106)	(0.109)
Manual			-0.300**	-0.300**	-0.248**
			(0.125)	(0.120)	(0.125)
MFP count (age 0-11)			-0.019	-0.025	-0.015
			(0.044)	(0.043)	(0.042)
Log average annual income FRS (age 0-11)			0.235**	0.240**	0.270**
			(0.109)	(0.107)	(0.109)
FDS age (0-11)			-0.022	-0.030	-0.016
			(0.047)	(0.045)	(0.046)
Mother has own house (age 11)			0.120	0.120	0.061
			(0.111)	(0.109)	(0.120)
**Children**					
BMI (age 15)				-0.042	-0.043
				(0.040)	(0.039)
Smoker (age 15)				-0.534***	-0.545***
				(0.161)	(0.161)
Drink alcohol (age 15)				-0.034	-0.014
				(0.078)	(0.079)
**Life events**					
Mother divorced (age 0-11)					-0.238
					(0.179)
Mother or partner cruel to child (age 0-11)					-0.038
					(0.125)
Diagnosed depression mother (age 12)					-0.078
					(0.129)
Mother has partner (age 12)					-0.223
					(0.209)
Any traumatic experience (age 0-10)					-0.114
					(0.108)
Observations	700	700	700	700	700
Adjusted R-squared	0.122	0.134	0.242	0.276	0.279

Notes: $^{*}p < 0.1$ $^{**}p < 0.05$, $^{***}p < 0.01$. The outcome variable is standardized, as are PoA and the following controls: birth weight, age of the mother at birth, MFP count, FDS, and BMI. For missing continuous variables the mean is taken and then the missing value is controlled for. Model 1: base and school fixed-effects controls; Model 2: base, pregnancy and school fixed-effects controls; Model 3: base, pregnancy, socioeconomic status and school fixed-effects controls; Model 4: base, pregnancy, socioeconomic status, children and school fixed-effects controls; Model 5: base, pregnancy, socioeconomic status, children, life events and school fixed-effects controls. School fixed-effects are not reported. Robust standard errors are reported in parentheses.

Table B.3: Gelbach decomposition based on PoA (age 15-19) estimates on KS4

	Base	Full	Explained
PoA (age 15-19)	-0.146***	-0.070*	-0.075***
	(0.043)	(0.039)	(0.025)
Pregnancy block			-0.005
			(0.007)
Socioeconomic status block			-0.044**
			(0.017)
Children block			-0.028**
			(0.012)
Life events block			0.002
			(0.008)

Notes: $^{*}p < 0.1$ $^{**}p < 0.05$, $^{***}p < 0.01$. The outcome variable is standardized, as are PoA and the following controls: birth weight, age of the mother at birth, MFP count, FDS, and BMI. The base specification and the full specification columns report the estimated correlation between PoA (age 15-19) and KS4 average grades, using base (including school fixed-effects) controls and the full battery of controls, respectively. The last column reports a decomposition of the difference between the estimated correlation in the base and in the full specifications, based on Gelbach (2016).

Table B.4: Descriptive statistics secondary analysis

	Type	Mean	Std. dev.	Min.	Max.	% Imputed
PoA (age 15-19)	Continuous	0.99	0.07	0.67	1.29	0
PoA (age 7-9)	Continuous	0.99	0.06	0.72	1.24	0
IQ (age 15)	Continuous	94.48	12.30	63	127	0
IQ (age 8)	Continuous	106.57	15.40	66	146	0
**Base**						
Age PoA (age 15-19)	Continuous	17.03	1.06	15	19.25	0
Age PoA (age 7-9)	Continuous	7.45	0.13	7.25	8.42	0
Age KS4	Continuous	16.23	0.31	15.75	16.67	0
Age KS1	Continuous	7.23	0.31	6.75	7.67	0
First born	Binary	0.43	.	.	.	1.16
Female	Binary	0.51	.	.	.	0
**Pregnancy**						
Folic acid intake	Binary	0.08	.	.	.	0.99
Caesarean	Binary	0.08	.	.	.	3.30
Smoke in pregnancy	Binary	0.14	.	.	.	8.91
Heavy drink in pregnancy	Binary	0.04	.	.	.	6.27
Birth weight (kg)	Continuous	3.50	0.49	1.49	5.14	1.49
**Socioeconomic status**						
Age of the mother at birth	Continuous	29.64	4.49	18	42	0.33
Maternal education:						
CSE, vocational, O-level	Binary	0.52	.	.	.	1.49
A-level	Binary	0.28	.	.	.	1.49
Degree	Binary	0.18	.	.	.	1.49
Paternal social class:						
Manual	Binary	0.36	.	.	.	6.93
Non-manual	Binary	0.42	.	.	.	6.93
Professional	Binary	0.15	.	.	.	6.93
MFP count (age 0-7)	Ordinal	0.72	1.30	0	6	1.16
MFP count (age 0-11)	Ordinal	0.83	1.45	0	8	0.17
Average annual income FRS (age 0-7)	Continuous	21.59	9.51	3.33	37.04	4.62
Average annual income FRS (age 0-11)	Continuous	23.84	9.68	3.11	47.93	1.65
Log average annual income FRS (age 0-7)	Continuous	2.95	0.52	1.20	3.61	4.62
Log average annual income FRS (age 7-11)	Continuous	3.07	0.47	1.13	3.87	1.65
FDS (age 0-7)	Continuous	2.53	2.73	0	12.33	1.16
FDS (age 0-11)	Continuous	2.24	2.38	0	11.75	3.80
Mother has own house (age 7)	Binary	0.83	.	.	.	0.33
Mother has own house (age 11)	Binary	0.83	.	.	.	0
**Children**						
BMI (age 7)	Continuous	16.26	2.14	12.65	29.15	0.33
BMI (age 15)	Continuous	21.45	3.67	14.70	41.63	0.99
Smoker (age 15)	Binary	0.09	.	.	.	2.31
Drink alcohol (age 15)	Binary	0.48	.	.	.	2.64
**Life events**						
Mother divorced (age 0-6)	Binary	0.05	.	.	.	19.64
Mother divorced (age 0-11)	Binary	0.09	.	.	.	24.75
Mother or partner cruel to child (0-6)	Binary	0.13	.	.	.	19.31
Mother or partner cruel to child (0-11)	Binary	0.16	.	.	.	23.93
Diagnosed depression mother (age 6)	Binary	0.08	.	.	.	9.08
Diagnosed depression mother (age 12)	Binary	0.08	.	.	.	13.37
Mother has partner (age 6)	Binary	0.87	.	.	.	8.75
Mother has partner (age 12)	Binary	0.82	.	.	.	12.05
Any traumatic experience (age 0-7)	Binary	0.07	.	.	.	9.24
Any traumatic experience (age 0-10)	Binary	0.14	.	.	.	9.24

Notes: Descriptive statistics are reported for the secondary estimation sample (606 observations). A description of all variables can be found in Appendix A. Only means (that can be interpreted as percentages) are reported for binary variables, as SD, Min and Max hold no statistical meaning for these variables. Imputed observations for binary, ordinal and categorical variables form a 'missing' category (not reported); imputed observations for continuous variables are set to be equal to the sample mean of the variable. 'KS1' and 'KS4' refer, respectively, to Key Stage 1 and Key Stage 4 average grades. As defined in Appendix A, 'MFP' stands for 'Major Financial Problem', 'FDS' is the 'Financial Difficulty Score', 'BMI' is the 'Body Mass Index', and 'FRS' refers to the 'Family Resources Survey'.

Table B.5: OLS estimates on IQ (age 8)

	model 1	model 2	model 3	model 4	model 5
PoA (7-9)	-0.059	-0.053	-0.021	-0.021	-0.025
	(0.041)	(0.042)	(0.039)	(0.039)	(0.039)
**Base**					
First born	0.063	0.102	0.061	0.065	0.037
	(0.082)	(0.086)	(0.084)	(0.084)	(0.085)
Age KS1	0.035	0.021	-0.003	0.004	-0.017
	(0.133)	(0.135)	(0.134)	(0.135)	(0.133)
Age PoA (age 7-9)	-3.201	-1.379	0.203	-0.359	0.419
	(15.963)	(16.633)	(16.242)	(15.631)	(14.488)
Age PoA squared (age 7-9)	0.172	0.061	-0.023	0.017	-0.026
	(1.052)	(1.097)	(1.073)	(1.031)	(0.955)
Female	-0.089	-0.077	-0.065	-0.059	-0.059
	(0.081)	(0.082)	(0.079)	(0.079)	(0.078)
**Pregnancy**					
Folic acid intake in pregnancy		0.110	0.054	0.060	0.058
		(0.161)	(0.147)	(0.148)	(0.143)
Caesarean		0.042	0.087	0.133	0.132
		(0.156)	(0.150)	(0.153)	(0.154)
Smoke in pregnancy		-0.143	0.115	0.128	0.126
		(0.115)	(0.118)	(0.118)	(0.116)
Heavy drink in pregnancy		0.220	0.054	0.059	0.049
		(0.192)	(0.198)	(0.199)	(0.197)
Birth weight		0.068	0.060	0.073*	0.061
		(0.042)	(0.039)	(0.040)	(0.040)
**Socioeconomic status**					
Age of the mother at birth			-0.007	-0.008	-0.013
			(0.046)	(0.046)	(0.044)
Maternal education:					
A-level			0.329***	0.325***	0.292***
			(0.091)	(0.091)	(0.093)
Degree			0.712***	0.694***	0.700***
			(0.122)	(0.122)	(0.122)
Paternal social class:					
Non-manual			-0.043	-0.019	0.007
			(0.121)	(0.122)	(0.119)
Manual			-0.310**	-0.286**	-0.233*
			(0.135)	(0.135)	(0.134)
MFP count (age 0-7)			0.049	0.051	0.055
			(0.046)	(0.046)	(0.046)
Log average annual income FRS (age 0-7)			0.161	0.168*	0.251**
			(0.099)	(0.099)	(0.100)
FDS (age 0-7)			0.052	0.052	0.070
			(0.054)	(0.054)	(0.055)
Mother has own house (age 7)			0.147	0.140	0.040
			(0.127)	(0.127)	(0.127)
**Children**					
BMI (age 7)				-0.064	-0.049
				(0.046)	(0.046)
**Life events**					
Mother divorced (age 0-6)					0.141
					(0.177)
Mother or partner cruel to child (age 0-6)					-0.117
					(0.141)
Diagnosed depression mother (age 6)					-0.144
					(0.126)
Mother has partner (age 6)					-0.353*
					(0.196)
Any traumatic experience (age 0-7)					-0.405***
					(0.142)
Observations	606	606	606	606	606
Adjusted R-squared	0.006	0.011	0.146	0.148	0.184

Notes: $^{*}p < 0.1$ $^{**}p < 0.05$, $^{***}p < 0.01$. The outcome variable is standardized, as are PoA and the following controls: birth weight, age of the mother at birth, MFP count, FDS, and BMI. For missing continuous variables the mean is taken and then the missing value is controlled for. For categorical variables a category is added for missing values. Model 1: base controls; Model 2: base, pregnancy controls; Model 3: base, pregnancy, socioeconomic status controls; Model 4: base, pregnancy, socioeconomic status, children controls; Model 5: base, pregnancy, socioeconomic status, children, life events controls. Robust standard errors are reported in parentheses.

Table B.6: OLS estimates on IQ (age 15)

	model 1	model 2	model 3	model 4	model 5
PoA (age 15-19)	-0.100**	-0.098**	-0.045	-0.044	-0.060
	(0.041)	(0.042)	(0.044)	(0.045)	(0.043)
**Base**					
First born	0.083	0.083	0.052	0.057	0.054
	(0.087)	(0.090)	(0.092)	(0.093)	(0.093)
Age KS4	0.128	0.090	0.066	0.086	0.097
	(0.147)	(0.148)	(0.149)	(0.150)	(0.153)
Age PoA (age 15-19)	-0.463	-0.700	-1.356	-1.843	-2.206
	(1.926)	(1.946)	(1.749)	(1.768)	(1.714)
Age PoA squared (age 15-19)	0.016	0.023	0.042	0.056	0.067
	(0.058)	(0.059)	(0.053)	(0.053)	(0.052)
Female	-0.230**	-0.240**	-0.254***	-0.253***	-0.237***
	(0.092)	(0.095)	(0.093)	(0.093)	(0.092)
**Pregnancy**					
Folic acid intake in pregnancy		0.181	0.169	0.234	0.249*
		(0.161)	(0.150)	(0.149)	(0.147)
Caesarean		-0.004	0.046	0.018	0.001
		(0.155)	(0.154)	(0.155)	(0.157)
Smoke in pregnancy		0.026	0.156	0.175	0.148
		(0.137)	(0.142)	(0.141)	(0.138)
Heavy drink in pregnancy		0.086	-0.002	-0.005	0.006
		(0.206)	(0.200)	(0.203)	(0.190)
Birth weight		-0.021	-0.028	-0.016	-0.021
		(0.045)	(0.043)	(0.043)	(0.042)
**Socioeconomic status**					
Age of the mother at birth			-0.019	-0.007	-0.014
			(0.056)	(0.055)	(0.055)
Maternal education:					
A-level			0.410***	0.411***	0.390***
			(0.101)	(0.102)	(0.101)
Degree			0.694***	0.697***	0.712***
			(0.151)	(0.151)	(0.151)
Paternal social class:					
Non-manual			-0.236	-0.246*	-0.195
			(0.144)	(0.141)	(0.142)
Manual			-0.374**	-0.372**	-0.327**
			(0.163)	(0.159)	(0.161)
MFP count (age 0-11)			0.032	0.031	0.032
			(0.049)	(0.049)	(0.050)
Log average annual income FRS (age 0-11)			0.160	0.159	0.231*
			(0.125)	(0.123)	(0.119)
FDS (age 0-11)			0.042	0.034	0.020
			(0.056)	(0.056)	(0.058)
Mother has own house (age 11)			0.058	0.060	-0.020
			(0.138)	(0.138)	(0.145)
**Children**					
BMI (age 15)				0.009	0.029
				(0.045)	(0.044)
Smoker (age 15)				-0.322**	-0.375***
				(0.140)	(0.138)
Drink alcohol (age 15)				0.024	0.030
				(0.088)	(0.089)
**Life events**					
Mother divorced (age 0-11)					0.184
					(0.173)
Mother or partner cruel to child (age 0-11)					0.019
					(0.130)
Diagnosed depression mother (age 12)					0.008
					(0.171)
Mother has partner (age 12)					-0.450**
					(0.212)
Any traumatic experience (age 0-10)					-0.288**
					(0.118)
Observations	606	606	606	606	606
Adjusted R-squared	0.095	0.090	0.192	0.207	0.233

Notes: $^{*}p < 0.1$ $^{**}p < 0.05$, $^{***}p < 0.01$. The outcome variable is standardized, as are PoA and the following controls: birth weight, age of the mother at birth, MFP count, FDS, and BMI. For missing continuous variables the mean is taken and then the missing value is controlled for. For categorical variables a category is added for missing values. Model 1: base and school fixed-effects controls; Model 2: base, pregnancy and school fixed-effects controls; Model 3: base, socioeconomic status and school fixed-effects controls; Model 4: base, pregnancy, socioeconomic status, children and school fixed-effects controls; Model 5: base, pregnancy, socioeconomic status, children, life events and school fixed-effects controls. School fixed-effects are not reported. Robust standard errors are reported in parentheses.

Table B.7: Gelbach decomposition based on PoA (age 15-19) estimates on IQ (age 15)

	Base	Full	Explained
PoA (age 15-19)	-0.100**	-0.060	-0.040
	(0.041)	(0.043)	(0.026)
Pregnancy block			0.005
			(0.010)
Socioeconomic status block			-0.059***
			(0.021)
Children block			-0.001
			(0.011)
Life events block			0.016
			(0.012)

Notes: $^{*}p < 0.1$ $^{**}p < 0.05$, $^{***}p < 0.01$. The outcome variable is standardized, as are PoA and the following controls: birth weight, age of the mother at birth, MFP count, FDS, and BMI. The base specification and the full specification columns report the estimated correlation between PoA (age 15-19) and IQ at age 15, using base (including school fixed-effects) controls and the full battery of controls, respectively. The last column reports a decomposition of the difference between the estimated correlation in the base and in the full specifications, based on Gelbach (2016).

Table B.8: Marginal effect of PoA (age 15-19), by contemporary PoA of the mother, on KS4 grades

PoA mother (child age 15-19)	model 1	model 2	model 3	model 4	model 5
-3	-0.026	0.009	0.087	0.065	0.061
	(0.122)	(0.121)	(0.112)	(0.110)	(0.110)
-2	-0.072	-0.044	0.022	0.017	0.013
	(0.090)	(0.088)	(0.082)	(0.081)	(0.081)
-1	-0.118*	-0.096	-0.044	-0.032	-0.036
	(0.061)	(0.060)	(0.056)	(0.055)	(0.055)
0	-0.164***	-0.148***	-0.109***	-0.080*	-0.084**
	(0.045)	(0.044)	(0.041)	(0.042)	(0.042)
1	-0.210***	-0.201***	-0.174***	-0.128**	-0.133**
	(0.053)	(0.053)	(0.050)	(0.052)	(0.051)
2	-0.256***	-0.253***	-0.240***	-0.177**	-0.182**
	(0.079)	(0.078)	(0.074)	(0.077)	(0.075)
3	-0.302***	-0.306***	-0.305***	-0.225**	-0.230**
	(0.111)	(0.110)	(0.104)	(0.106)	(0.104)

Notes: $^{*}p < 0.1$ $^{**}p < 0.05$, $^{***}p < 0.01$. Both the mother's and child's PoA are standardized. Model 1: Base, school controls; Model 2: Base, school, pregnancy controls; Model 3: Base, school, pregnancy, socioeconomic status controls; Model 4: Base, school, pregnancy, socioeconomic status, children controls; Model 5: Base, school, pregnancy, socioeconomic status, children, life events controls. Robust standard errors are reported in parentheses. Marginal effects are based on Table B.10.

Table B.9: Marginal effect of PoA (age 15-19), by contemporary PoA of the mother, on IQ (age 15)

PoA mother (child age 15-19)	model 1	model 2	model 3	model 4	model 5
-3	-0.091	-0.066	0.036	0.010	0.026
	(0.115)	(0.117)	(0.129)	(0.130)	(0.126)
-2	-0.103	-0.087	-0.001	-0.016	-0.011
	(0.085)	(0.087)	(0.097)	(0.097)	(0.094)
-1	-0.115*	-0.108*	-0.037	-0.042	-0.047
	(0.059)	(0.061)	(0.068)	(0.069)	(0.067)
0	-0.128***	-0.129***	-0.074	-0.068	-0.084*
	(0.046)	(0.047)	(0.050)	(0.051)	(0.050)
1	-0.140**	-0.150***	-0.111**	-0.095*	-0.121**
	(0.055)	(0.056)	(0.054)	(0.057)	(0.055)
2	-0.152*	-0.171**	-0.147*	-0.121	-0.157**
	(0.079)	(0.080)	(0.077)	(0.081)	(0.078)
3	-0.164	-0.192*	-0.184*	-0.147	-0.194*
	(0.109)	(0.110)	(0.107)	(0.112)	(0.108)

Notes: $^{*}p < 0.1$ $^{**}p < 0.05$, $^{***}p < 0.01$. Both the mother's and child's PoA are standardized. Model 1: Base, school controls; Model 2: Base, school, pregnancy controls; Model 3: Base, school, pregnancy, socioeconomic status controls; Model 4: Base, school, pregnancy, socioeconomic status, children controls; Model 5: Base, school, pregnancy, socioeconomic status, children, life events controls. Robust standard errors are reported in parentheses. Marginal effects are based on Table B.11.

Table B.10: OLS estimates on KS4 (age 15-17): mother child PoA interaction

	model 1	model 2	model 3	model 4	model 5
PoA (age 15-19)	-0.164***	-0.148***	-0.109***	-0.080*	-0.084**
	(0.045)	(0.044)	(0.041)	(0.042)	(0.042)
PoA mother (age child 15-19)	0.031	0.057	0.043	0.042	0.037
	(0.040)	(0.040)	(0.039)	(0.039)	(0.039)
PoA × PoA mother (age child 15-19)	-0.046	-0.052	-0.065**	-0.048	-0.049
	(0.036)	(0.035)	(0.033)	(0.033)	(0.033)
**Base**					
First born	0.141*	0.150*	0.115	0.096	0.088
	(0.080)	(0.080)	(0.084)	(0.083)	(0.084)
Age KS4	0.121	0.109	0.173	0.183	0.140
	(0.133)	(0.133)	(0.129)	(0.127)	(0.129)
Age PoA (age 15-19)	-0.052	-0.566	-0.090	-0.258	-0.364
	(1.701)	(1.702)	(1.577)	(1.571)	(1.570)
Age PoA squared (age 15-19)	0.005	0.020	0.005	0.009	0.013
	(0.051)	(0.051)	(0.047)	(0.047)	(0.047)
Female	0.228***	0.220***	0.210***	0.219***	0.223***
	(0.082)	(0.082)	(0.078)	(0.077)	(0.076)
**Pregnancy**					
Folic acid intake in pregnancy		-0.056	-0.023	0.017	0.055
		(0.138)	(0.128)	(0.125)	(0.125)
Caesarean		0.043	0.091	0.142	0.161
		(0.155)	(0.147)	(0.150)	(0.150)
Smoke in pregnancy		-0.395***	-0.199	-0.159	-0.167
		(0.134)	(0.129)	(0.130)	(0.128)
Heavy drink in pregnancy		-0.072	-0.162	-0.130	-0.132
		(0.205)	(0.218)	(0.213)	(0.214)
Birth weight		0.015	0.006	0.027	0.027
		(0.042)	(0.040)	(0.040)	(0.040)
**Socioeconomic status**					
Age of the mother at birth			0.003	0.003	-0.010
			(0.046)	(0.045)	(0.045)
Maternal education:					
A-level			0.245**	0.222**	0.234**
			(0.098)	(0.097)	(0.098)
Degree			0.533***	0.511***	0.545***
			(0.132)	(0.132)	(0.134)
Paternal social class:					
Non-manual			0.027	0.018	0.051
			(0.111)	(0.109)	(0.113)
Manual			-0.263**	-0.263**	-0.210
			(0.128)	(0.124)	(0.129)
MFP count (age 0-11)			-0.019	-0.024	-0.016
			(0.044)	(0.043)	(0.042)
Log average annual income FRS (age 0-11)			0.226**	0.235**	0.265**
			(0.110)	(0.109)	(0.112)
FDS (age 0-11)			-0.020	-0.030	-0.015
			(0.048)	(0.047)	(0.048)
Mother has own house (age 11)			0.103	0.113	0.031
			(0.111)	(0.111)	(0.125)
**Children**					
BMI (age 15)				-0.049	-0.051
				(0.041)	(0.041)
Smoker (age 15)				-0.481***	-0.499***
				(0.162)	(0.163)
Drink alcohol (age 15)				-0.071	-0.053
				(0.080)	(0.080)
**Life events**					
Mother divorced (age 0-11)					-0.215
					(0.182)
Mother or partner cruel to child (age 0-11)					-0.014
					(0.131)
Diagnosed depression mother (age 12)					-0.098
					(0.136)
Mother has partner (age 12)					-0.216
					(0.215)
Any traumatic experience (age 0-10)					-0.119
					(0.107)
Observations	666	666	666	666	666
Adjusted R-squared	0.127	0.139	0.243	0.274	0.276

Notes: $^{*}p < 0.1$ $^{**}p < 0.05$, $^{***}p < 0.01$. The outcome variable is standardized, as are child's and mother's PoA and the following controls: birth weight, age of the mother at birth, MFP count, FDS, and BMI. For missing continuous variables the mean is taken and then the missing value is controlled for. Model 1: base and school fixed-effects controls; Model 2: base, pregnancy and school fixed-effects controls; Model 3: base, pregnancy, socioeconomic status and school fixed-effects controls; Model 4: base, pregnancy, socioeconomic status, children and school fixed-effects controls; Model 5: base, pregnancy, socioeconomic status, children, life events and school fixed-effects controls. School fixed-effects are not reported. Robust standard errors are reported in parentheses.

Table B.11: OLS estimates on IQ (age 15): mother child PoA interaction

	model 1	model 2	model 3	model 4	model 5
PoA (age 15-19)	-0.128***	-0.129***	-0.074	-0.068	-0.084*
	(0.046)	(0.047)	(0.050)	(0.051)	(0.050)
PoA mother (age of child 15-19)	0.050	0.056	0.046	0.047	0.045
	(0.041)	(0.043)	(0.042)	(0.042)	(0.041)
PoA × PoA mother (age of child 15-19)	-0.012	-0.021	-0.037	-0.026	-0.037
	(0.034)	(0.034)	(0.036)	(0.037)	(0.035)
**Base**					
First born	0.085	0.089	0.036	0.040	0.043
	(0.090)	(0.093)	(0.096)	(0.098)	(0.099)
Age KS4	0.134	0.094	0.096	0.126	0.117
	(0.151)	(0.151)	(0.152)	(0.152)	(0.155)
Age PoA (age 15-19)	-0.806	-1.149	-1.707	-2.061	-2.483
	(1.944)	(1.971)	(1.763)	(1.787)	(1.733)
Age PoA squared (age 15-19)	0.026	0.036	0.053	0.063	0.076
	(0.058)	(0.059)	(0.053)	(0.054)	(0.052)
Female	-0.229**	-0.242**	-0.271***	-0.265***	-0.246**
	(0.095)	(0.098)	(0.096)	(0.096)	(0.096)
**Pregnancy**					
Folic acid intake in pregnancy		0.253	0.257*	0.322**	0.343**
		(0.167)	(0.152)	(0.152)	(0.151)
Caesarean		-0.022	0.025	0.010	-0.014
		(0.162)	(0.162)	(0.164)	(0.166)
Smoke in pregnancy		-0.031	0.093	0.111	0.086
		(0.143)	(0.145)	(0.147)	(0.140)
Heavy drink in pregnancy		0.068	-0.019	-0.007	-0.011
		(0.219)	(0.211)	(0.213)	(0.198)
Birth weight		-0.015	-0.030	-0.017	-0.023
		(0.047)	(0.045)	(0.045)	(0.044)
**Socioeconomic status**					
Age of the mother at birth			-0.045	-0.037	-0.051
			(0.057)	(0.056)	(0.056)
Maternal education:					
A-level			0.434***	0.430***	0.420***
			(0.105)	(0.107)	(0.106)
Degree			0.702***	0.708***	0.735***
			(0.153)	(0.153)	(0.154)
Paternal social class:					
Non-manual			-0.244*	-0.250*	-0.193
			(0.144)	(0.142)	(0.145)
Manual			-0.382**	-0.396**	-0.341**
			(0.165)	(0.163)	(0.166)
MFP count (age 0-11)			0.027	0.026	0.021
			(0.051)	(0.050)	(0.052)
Log average annual income FRS (age 0-11)			0.172	0.167	0.243**
			(0.126)	(0.124)	(0.121)
FDS (age 0-11)			0.042	0.034	0.024
			(0.058)	(0.057)	(0.059)
Mother has own house (age 11)			0.049	0.059	-0.039
			(0.142)	(0.142)	(0.149)
**Children**					
BMI (age 15)				0.001	0.019
				(0.047)	(0.045)
Smoker (age 15)				-0.319**	-0.375***
				(0.145)	(0.143)
Drink alcohol (age 15)				0.023	0.035
				(0.093)	(0.094)
**Life events**					
Mother divorced (age 0-11)					0.173
					(0.175)
Mother or partner cruel to child (age 0-11)					0.089
					(0.135)
Diagnosed depression mother (age 12)					-0.099
					(0.173)
Mother has partner (age 12)					-0.450**
					(0.221)
Any traumatic experience (age 0-10)					-0.291**
					(0.123)
Observations	577	577	577	577	577
Adjusted R-squared	0.092	0.089	0.194	0.205	0.232

Notes: $^{*}p < 0.1$ $^{**}p < 0.05$, $^{***}p < 0.01$. The outcome variable is standardized, as are child's and mother's PoA and the following controls: birth weight, age of the mother at birth, MFP count, FDS, and BMI. For missing continuous variables the mean is taken and then the missing value is controlled for. Model 1: base and school fixed-effects controls; Model 2: base, pregnancy and school fixed-effects controls; Model 3: base, pregnancy, socioeconomic status and school fixed-effects controls; Model 4: base, pregnancy, socioeconomic status, children and school fixed-effects controls; Model 5: base, pregnancy, socioeconomic status, children, life events and school fixed-effects controls. School fixed-effects are not reported. Robust standard errors are reported in parentheses.
